# Biodegradation of PHBV-based biocomposites in two different marine environments of the Mediterranean Sea

**DOI:** 10.1007/s10532-025-10231-4

**Published:** 2025-12-09

**Authors:** Patricia Feijoo, Anna Marín, José Tena-Medialdea, José Rafael García-March, José Gámez-Pérez, Luis Cabedo

**Affiliations:** 1 Polymers and Advanced Materials Group (PIMA), Universitat Jaume I (UJI), Avenida Vicent Sos Baynat S/N, 12071 Castelló, Spain; 2 IMEDMAR-UCV-Institute of Environment and Marine Science Research, Universidad Católica de Valencia, C. Explanada del Puerto S/N, Calpe, 03710 Alicante, Spain; 3 CEBIMAT LAB S.L, ESPAITEC, Universitat Jaume I, Av. Vicent Sos Baynat S/N, 12071 Castelló, Spain

**Keywords:** Marine biodegradation, Biopolymers, Natural fibers, Open environment biodegradation, Polyhydroxyalkanoates, Biocomposites

## Abstract

Plastic pollution has become one of the most pressing environmental issues worldwide, with large amounts of conventional plastics accumulating in terrestrial and marine ecosystems due to their persistence and ineffective waste management. Developing and understanding the biodegradation behavior of environmentally friendly alternatives, such as bioplastics, is therefore crucial to mitigate this problem. In this context, the degradation of PHBV-based biocomposites containing purified cellulose (TC), wood flour (WF), and almond shell (AS) fibers have been investigated and compared with neat PHBV in two Mediterranean marine locations—a port and the open sea, within the same geographic region. Changes in weight, surface morphology, surface roughness, surface chemistry, and mechanical properties were monitored and periodically evaluated over 18 months of seawater exposure at the two sites. After 18 months of immersion, PHBV/AS showed the highest disintegration degree (88% for 150 µm films and 33% for 500 µm sheets), with the port environment promoting up to a two- to three-fold higher biodegradation rate compared to the open sea. Additionally, mineralization was studied in lab-simulated marine conditions by tracking CO_2_ release in order to study the actual effect of the fibers on the biodegradation rate of the PHBV. The research highlighted the significant influence of habitat-specific factors on biodegradation, with the port environment exhibiting a more pronounced impact on bacterial adhesion, weight loss, and the deterioration of mechanical properties compared to the open sea. Lignocellulosic fillers, regardless of type, promoted PHBV biodegradation in both conditions. In particular, PHBV/AS exhibited the highest disintegration degree, followed by PHBV/TC and PHBV/WF. Fiber characteristics such as size, shape, and porosity predominantly governed biocomposite disintegrability. Almond shell was revealed as the most favorable fiber for PHBV biodegradation during mineralization test. Under laboratory-simulated marine conditions, the composites reached 50% mineralization between 55 and 70% faster than neat PHBV, confirming the accelerating effect of the fibers on the biodegradation kinetics. This study aims to shed light on the understanding of the biodegradation mechanism of biodegradable polymers and the effect of cellulosic fillers on this natural process. Additionally, the study includes tests and measurements of biodegradation under real conditions, which will provide further insights into the kinetics of this process. This knowledge is of interest for designing biodegradable products and predicting their biodegradation time.

## Introduction

Polymers shape our world in thousands of plastic products that bring convenience to our daily lives. These lightweight, strong, versatile, and moldable materials are massively produced mainly for short-term applications (e.g. bags, cutlery, cups, bottles, packaging) (Wei et al. 2015). Besides, their long life as a residue along with an inefficient waste management system, has led the world to a global problem of plastic pollution (Wei et al. 2015; Rhodes 2018). From the total amount of plastic produced since 1950, around 9% has been recycled, 12% has been incinerated, and the remaining 79% either has been stored in landfills or has been released (unintentionally, we should believe) into the natural environment (Rhodes 2018). One of the most threatened ecosystems is the ocean, in which every year 5 to 13 million tons of plastic waste enter and accumulate as debris (Wang et al. 2021). Actually, marine plastic waste pollution has been listed as one of the top ten environmental problems to be solved globally since the first UN Environment Conference in 2014 (Wang et al. 2021). The existing measures are focused on the triple R – reduce, reuse and recycle – (Wang et al. 2021), while the scientific community is making efforts to develop more sustainable materials for the future (Wei et al. 2015).

In this sense, polyhydroxyalkanoates (PHA) have gained great interest as environmentally friendly materials. PHA are a family of microbial polyesters well known for their bio-based and potentially renewable origin (Koller et al. 2017), and their biodegradable character in a whole range of aerobic and anaerobic environments (e.g. compost, soil, water, landfill) (Meereboer et al. 2020a; Bátori et al. 2018; Dilkes-Hoffman et al. 2019). In particular, poly(3-hydroxybutyrate-co-3-hydroxyvalerate) (PHBV) is one of the most promising PHA to truly gain a foothold in the market due to its similar mechanical performance to some conventional thermoplastics like polypropylene (PP) (Rivera-Briso and Serrano-Aroca 2018). However, its extensive presence in the market will be restricted until overcoming some applicability weaknesses like high intrinsic fragility and narrow processing window (Wei et al. 2015; Sánchez-Safont et al. 2018), as well as the economic issue of its 4.5 times higher production cost compared to traditional polymers (Sánchez-Safont et al. 2018). A common strategy to reduce its high price is to replace a part of PHBV with low-cost fillers like abundantly available plant-based fibers (Rangappa et al. 2022), always looking for synergy effects in its mechanical properties (Rivera-Briso and Serrano-Aroca 2018; Hammiche et al. 2020). For instance, the three short fibers (flax, hemp and wood) selected by Fracz et al. (Fracz et al. 2021) to be blended with PHBV in a 30% w/w ratio, improved Young’s modulus by more than 150%, being the best results achieved with hemp fiber. When Singh et al. (Singh et al. 2008) added 40% w/w of maple wood fiber, the tensile and flexural modulus of PHBV was improved by 167% and the deflection temperature was increased by 21%. With the addition of 60% w/w luffa fiber, Guo et al. (Guo et al. 2019) improved flexural strength by 220% with respect to pure PHBV. The performance of lignocellulose-loaded composites can be further improved by enhancing interfacial adhesion with the matrix. Alkali treatment of the fiber to remove lignin, pectin, and waxes modifies the polarity character and thus, water sorption (Sánchez-Safont et al. 2018) while grafting changes the molecular structure of both fiber and polyester and thus, covalent bonding between them (Wei et al. 2015). These chemical modifications could vary the biodegradability of PHA in a synergistic or antagonistic way (Meereboer et al. 2020a).

Degradation of PHA starts with the fragmentation of the polymer into small pieces through two simultaneous ways: 1) abiotic or physical–chemical degradation, which breaks down the polymer by the effect of weathering, UV-irradiation, mechanical forces, abiotic hydrolysis and so forth (Wang et al. 2021; Hammiche et al. 2020), and 2) biotic or biodegradation, which after the formation of a biofilm on the surface of the material, proceeds via enzymatic hydrolysis of the ester bonds by the action of extracellular depolymerases secreted by the microorganisms attached (Wang et al. 2021; Dilkes-Hoffman et al. 2019). Eventually, it leads to the formation of soluble oligomers, dimers, and monomers that can enter the bacterial cell membrane (bioassimilation) to be used as carbon and energy sources, resulting in simple end-products like CO_2_ and H_2_O (mineralization) (Wang et al. 2021). As bioassimilation and mineralization are assumed to be rapid (Dilkes-Hoffman et al. 2019; Chinaglia et al. 2018), enzymatic hydrolysis is considered the rate-limiting step in biodegradation (Wang et al. 2021). The rate of biodegradation of PHA is highly dependent on intrinsic features of the polyester such as molecular weight, crystallinity, or hydrophobic character, which all may hamper the penetration of water and enzymes into the inner structure, resulting in slower hydrolysis (Emadian et al. 2017; Silva et al. xxxx). Other influential parameters are the size and shape of the material, that is, surface contact area with the environment (Wang et al. 2021; Emadian et al. 2017). For instance, in lab-simulated marine conditions, PHBV microbeads reached 17% mass loss in 2 months (Cheng et al. 2022) while it was reported only an 8% weight loss in 12 months in the case of 4 mm dog-bone specimens (Deroiné et al. 2015).

In general, the presence of fibers has been reported (and in many cases, assumed) to promote PHA biodegradation (Meereboer et al. 2020a). Their effect depends on fiber content, chemical composition, hydrophilicity (water sorption), size and morphology of the particles (interfacial area fiber-matrix) (Silva et al. xxxx). In this regard, recent research on PLA-based composites reinforced with three different natural fibers demonstrated that fiber porosity plays a decisive role in the photochemical degradation of the polymer matrix in marine environment (Maio et al. 2025). Although these studies have contributed to a better understanding of the relationship between fiber characteristics and degradation behavior, most of them were conducted with other polymer matrices, under terrestrial or controlled laboratory conditions (Hubbe et al. 2020; Muniyasamy et al. 2013), therefore literature with data on their biodegradation in marine environments is relatively scarce. Moreover, this research has been very disparate in terms of results, settings and conditions. Seggiani et al. (Seggiani et al. 2018) found 23% mass loss of PHBV/Posidonia oceanica (80/20) pellets in natural marine mesocosm after 1 year of immersion and 25% of mineralization of the same material in lab-simulated marine conditions following ASTM D 6691 standard. By contrast, Meereboer et al. (Meereboer et al. 2021) evaluated the biodegradation of PHBV/Miscanthus (75/25) in powder form following ASTM D 7991 and reported complete mineralization of the composite in 412 days. At present, there is a lack of consensus in lab testing methods to determine marine biodegradation of plastics and composites.

Therefore, it is still necessary more research to mimic biological, physical, and chemical conditions of the complex marine environment (Cheng et al. 2022), composed of a great variety of habitats (supralittoral, eulittoral, sublittoral benthic, deep sea benthic, pelagic and buried in the sediments) (Emadian et al. 2017; Cheng et al. 2022). Besides, each specific habitat presents different characteristics such as temperature, nutrient profile, UV light, dissolved oxygen, strength of mechanical forces or the abundance of degrading microbes, that may influence the biodegradation rate (Wang et al. 2021; Suzuki et al. 2021). More studies in natural marine environments are necessary to converge results in order to develop lifetime prediction systems for biocomposites in the ocean, similar to the meta-study for PHA ran by Dilkes-Hoffman et al. (Dilkes-Hoffman et al. 2019).

Within this context, the main objective of this work is to analyze the effect on the marine biodegradability of PHBV-based biocomposites of three different organic fillers (cellulose, wood flour, almond shell) in two different marine habitats (open sea and port). Over 18 months of immersion in both natural environments, the progress of biodegradation was monitored by mass loss, microscopy, and infrared spectroscopy. Changes in mechanical properties, surface roughness, and microbial population were also assessed. In addition, mineralization test was performed in lab-simulated marine conditions following a modification of the standard ASTM D6691. Overall, this research constitutes an extensive and thorough long-term study on the biodegradation of lignocellulose-PHBV biocomposites in marine environments.

## Materials and methods

### Materials

**Fig. 1:**
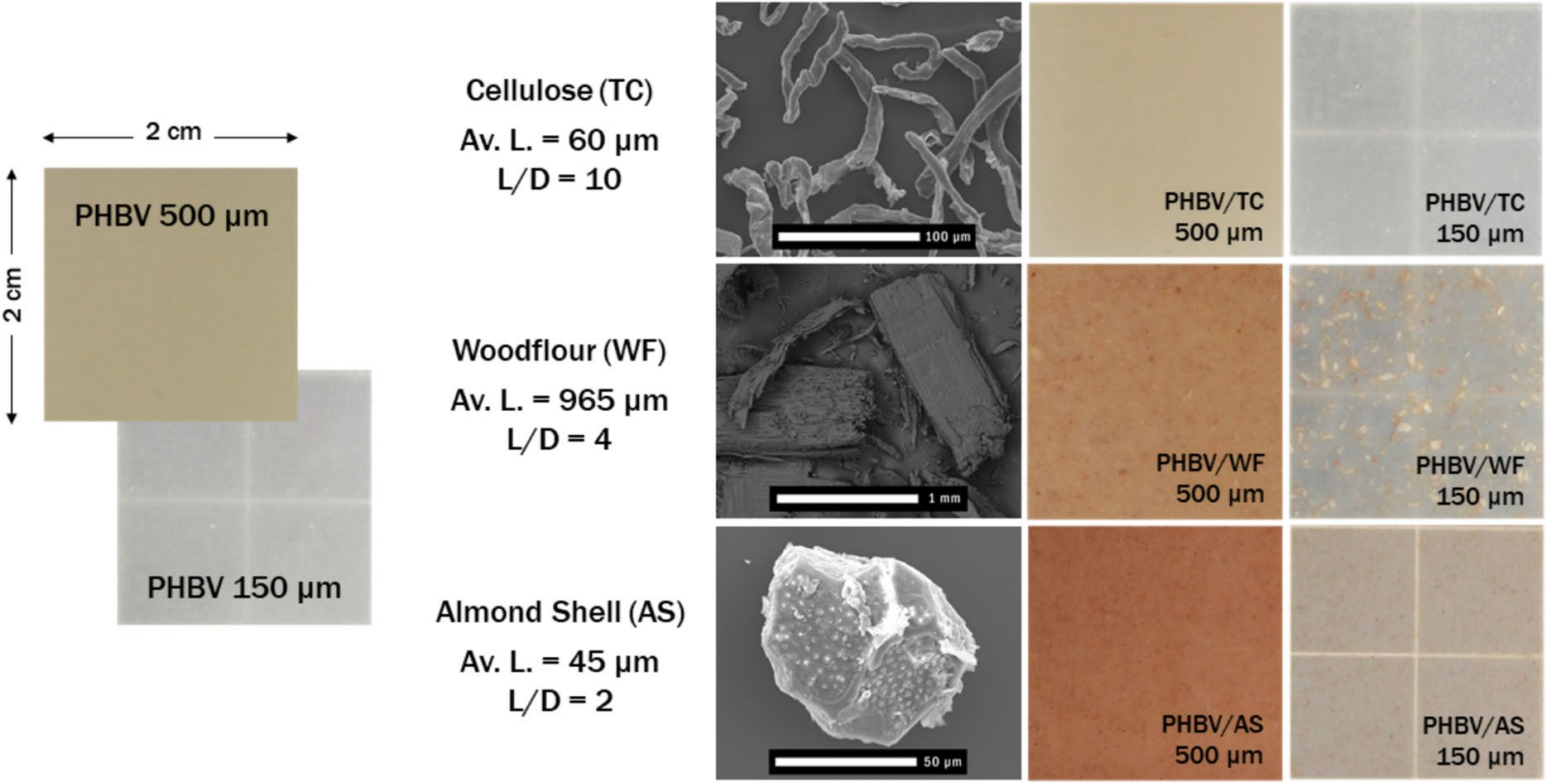
Visual appearance of films and sheets of the samples studied, as well as SEM micrographs of fillers used in biocomposites. Average length (Av.L.) and aspect ratio (L/D) of fibers are detailed

Pelletized PHBV, commercial grade ENMAT Y1000P with 3 wt.% of 3-hydroxyvalerate, was supplied by Tianan Biologic Material Co. (China). Three different fiber-like fillers were considered for the composites: purified alpha-cellulose TC90 (TC) (bar-shaped fibrils with a smooth surface, average length = 60 µm, aspect ratio L/D = 10) from CreaFill Fibers Corp. (Chestertown, USA), pine tree wood flour (WF) (rectangular-shaped chips with rough and cracked surface, average length = 965 µm, aspect ratio L/D = 4) from J.Adrian S.L. (Burjassot, Spain), and micronized almond shell (AS) (round-shaped particles with a porous surface full of pinholes, average length = 45 µm, aspect ratio L/D = 2) kindly supplied by Unió Corporació Alimentària (Reus, Spain). The appearance of the fillers used can be observed in Fig. 1. For language simplicity, all three will be referred from now on as fibers. All culture media used for microbiological characterization were provided by Laboratorios Conda S.A (Madrid, Spain). Antibiotic and antifungal compounds were acquired from Merck Life Science S.L. (Madrid, Spain) as well as other common reagents used (NaCl, KCl, Na_2_HPO_4_ and KH_2_PO_4_).

### Sample preparation

Neat PHBV and three biocomposites were studied and named as follows: PHBV with 15% wt. of pure cellulose (PHBV/TC), PHBV with 15% wt. of wood flour (PHBV/WF), and PHBV with 10 phr (9.1% equivalent) of almond shell (PHBV/AS). This last formulation was selected from a previous European project (YPACK) due to its similar composition as well as the presence of a different and interesting agro-waste as filler. As detailed below, the four (4) samples were obtained in 500 and 150 µm films.

### Compounding

Previously to compounding, PHBV and fillers (TC90, WF, AS) were dried at 80 °C for at least 24 h. The components of each formulation were manually dry-mixed and directly fed in the hopper of the extruder. PHBV/TC and PHBV/WF biocomposites were produced in a co-rotating twin-screw extruder (Haisin TSE-20B, Nanjing, China), at a constant speed of 200 rpm and a temperature profile of 175/173/165/165/165/165/165 °C from hopper to nozzle. PHBV/AS was produced at the compounding facilities of Universidade do Minho (UMINHO, Braga, Portugal) using a reverse temperature profile in a twin screw co-rotating extruder at 100 rpm. The threads obtained were cooled down in a water bath and subsequently pelletized.

### Cast extrusion (500 µm)

Pellets were dried at 80 °C for at least 4 h in a dehumidifier (Piovan DPA 10, Santa Maria di Sala, Italy) and cast extruded into sheets of 500 µm nominal thickness using a laboratory scale single-screw extruder (Rheomix 3000P ThermoHaake, Karlsruhe, Germany) with a calendering system. The temperature profile was set at 145/165/175/175 °C from hopper to fishtail nozzle and the rotation speed of the screw was set at 120 rpm.

### Compression molding (150 µm)

Additionally, cast extruded pieces of 4 × 4 cm were compressed in a hot-plate hydraulic press (Carver 4122, USA) to obtain films of 150 µm nominal thickness. All samples were processed at 180 °C of temperature and 5 bar of pressure for 3 min. The visual appearance of the materials obtained can be observed in Fig. 1.

### Set up and exposure to the marine environment

**Fig. 2:**
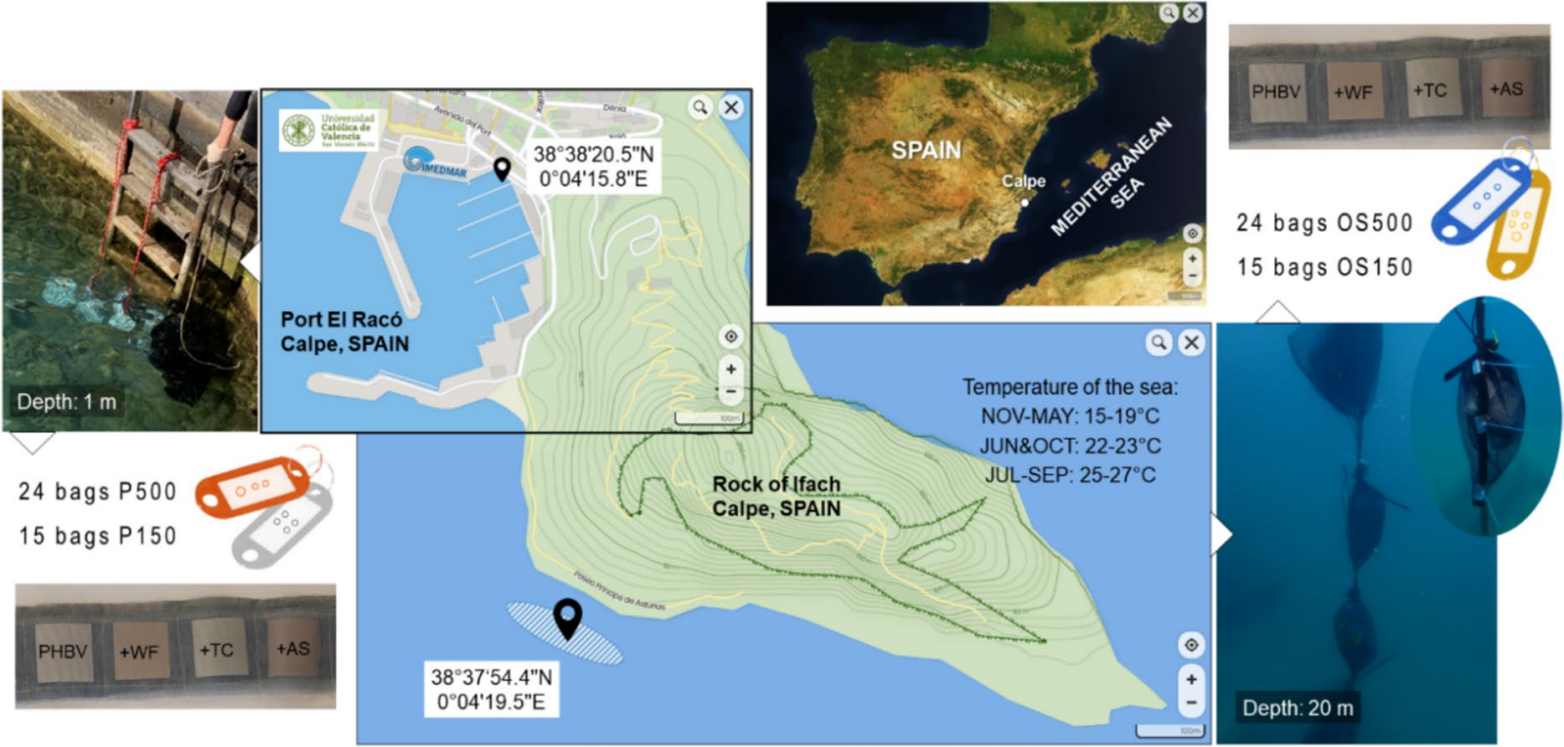
Maps, locations, and experimental setup

Each material film of both thicknesses (500 and 150 µm) was cut into pieces of 4 × 4 cm, dried at 40 °C under vacuum for 24 h, and weighed. One piece per material was inserted into a custom-made 1 mm-mesh nylon bag. Each bag was numbered by a plastic-holed label. Bags were half-divided and exposed to two different marine environments close to Calpe (Alicante, Spain): the fishing port (P) and the open sea (OS) near Rock of Ifach. Setup and specific locations are summarized in Fig. 2. To sum up, four different conditions can be distinguished for each material: P500, OS500, P150, OS150.

Two replicates of each material and thickness (2 of each color label) were randomly collected after 1, 2, 4, 5, 6, 8, 10, 12, and 18 months of exposure, starting September 2020 to March 2022. Nylon bags were transported to the Institute of Environment and Marine Science Research (Calpe, Spain) where they were stored at 4 °C and sent to the Polymers and Advanced Materials Laboratory (Castelló, Spain) to be analyzed within a maximum of 24 h time range. The effect on biodegradation of PHBV of the specific environment (port or open sea) and the type of filler were studied. For that purpose, changes in mass, roughness, surface morphology, chemical structure, microbial dynamics, and mechanical properties were analyzed. Additionally, results were complemented with a mineralization study in lab-simulated marine conditions.

### Colonization of the surface: enumeration of cultivable marine bacteria

Due to their higher surface integrity, biofilm was analyzed only on 500 µm samples. In the case of samples located in port (P) environment, this was done only up to month 6. Each piece was scraped with a sterile scalpel blade and the detached biofilm was placed in a Falcon tube containing 20 mL of phosphate-buffered saline (NaCl 8 g/L, KCl 0.2 g/L, Na_2_HPO_4_ 1.4 g/L, KH_2_PO_4_ 0.2 g/L). After vortexing for 1 min, suspensions were serially diluted by duplicate and spread on marine agar (MA) for the quantification of cultivable heterotrophic marine bacteria. The medium was supplemented with cycloheximide (0.01 g/L) to suppress molds growth. The plates were incubated at 26 °C for 4 days and colony forming units (CFU) were counted. Results were expressed as log CFU/cm^2^ material. Analysis of variance (ANOVA) was applied and significant differences were determined with the least significant difference (LSD) test (*p* < 0.05) using Statgraphics Centurion XVI version 16.1.17 (Manugistics Corp. Rockville, MD, USA).

### Surface roughness and mass loss

The integrity of the materials over time was assessed by monitoring the changes in weight and roughness. Pieces were washed in distilled water and dried at 40 °C under vacuum for 24 h prior to analysis.

#### Scanning electron microscopy (SEM)

The morphology of the surface was studied by SEM. Micrographs were taken by a JEOL 7001F microscope at a voltage of 5 kV. Prior to observation, samples were dried under vacuum and coated with Pt by sputtering deposition under an argon atmosphere for 15 s (BalTec500, Switzerland).

#### Optical profilometry

The surface topography (2D and 3D images) and roughness parameters (Pa, Pv, Pp and Pt) were analyzed by a PLµ 2300 optical profiler (Sensofar, Terrasa, Spain) with a magnification of 50x. Per sample, five zones of 255 × 190 µm^2^ were measured. Pa parameter is the arithmetic mean of the height of a limited area, Pv and Pp are the highest and the lowest height of the roughness profile respectively, and Pt is the difference between Pv and Pp.

#### Disintegration

The disintegration degree (D%) was calculated by subtracting the mass of each piece at every time of exposure from its initial mass using the Eq. 1:

1 $$D\left(\%\right)= \left(\frac{{m}_{i}- {m}_{t}}{{m}_{i}}\right)\cdot 100$$

where mi is the initial dry mass of a piece and mt is the dry mass of the same piece at a specific time of exposure. The disintegration degree of a material was reported as the average value of its pieces.

### Surface chemistry: fourier-transform infrared spectroscopy (ATR-FTIR)

Chemical changes over degradation time were studied by FTIR spectroscopy. The spectra of the materials were collected by a Jasco FT/IR-6200 (Madrid, Spain) equipped with an attenuated total reflection (ATR) accessory in the range of 400–4000 cm^−1^ in transmission mode. For better comparison of degradation progress, the spectra were normalized using the peak at 1130 cm^−1^ (C–O–C stretching) as an internal standard.

#### Structural deterioration: mechanical properties

For 500 µm materials, tensile tests were carried out in a universal testing machine (Shimadzu, Japan) at a cross-speed of 1 mm/min and a gap between clamps of 15 mm. Elastic modulus, tensile strength, and elongation at break were determined. For 150 µm materials, due to their fragility, tensile tests were performed in a Discovery DHR-1 hybrid rheometer (TA Instruments, New Castle, USA) equipped with a clamp system for dynamic mechanical analysis (DMA) in tensile mode. In both cases, rectangular specimens of 6 mm wide and 35 mm length were used.

#### Biodegradation in simulated lab conditions

The ultimate biodegradation (or mineralization) test was carried out following a modified method based on the standard ASTM D6691 (ASTM D6691–17. xxxx). Airtight 500 ml-bioreactors customized for respirometry were used. Per reactor, 60 mg of powdered sample (< 250 µm) were mixed with 225 ml of autoclaved seawater from Calpe (Alicante, Spain) supplemented with 0.5 g/L NH_4_Cl and 0.1 g/L KH_2_PO_4_. An enriched inoculum obtained from biofilms formed on the surface of PHBV samples exposed to the seawater of Calpe’s port was inoculated in each reactor at an initial concentration of 2·10^4^ CFU/ml. The four materials plus a blank were tested in triplicate. Bioreactors were kept at 26 °C and a rotation of 50 rpm for ca.300 days in an orbital shaking incubator (Comecta model 2102, Barcelona, Spain). Carbon dioxide evolved by microorganisms was registered by a LI-850 CO_2_ gas analyzer (LI·COR, Madrid, Spain). The measurement frequency was established based on the pH of the water, which slows down or stops the microbial activity when reaches an acid range (Das and Mangwani 2015; Ho et al. 2002). Aerobic conditions were guaranteed throughout the entire experiment by oxygenation with new air after each measurement.

Relative biodegradation (RB%) was reported as the average value of the replicates calculated by the Eq. 2:

2 $$RB\left(\%\right)= \left(\frac{{CO}_{2 }\left(t\right)- {CO}_{2} (b)}{{CO}_{2 }\left(f\right)- {CO}_{2} (bf)}\right)\cdot 100$$

where CO_2_ (t) is the accumulated carbon dioxide of a sample replicate at a specific time, CO_2_ (b) is the average accumulated carbon dioxide of the blank at the same time, CO_2_ (f) is total the accumulated carbon dioxide of a sample replicate at the end of the experiment, CO_2_ (bf) is the average accumulated carbon dioxide of the blank at the end of the experiment.

## Results and discussion

### Colonization of the surface: enumeration of cultivable marine bacteria

**Fig. 3:**
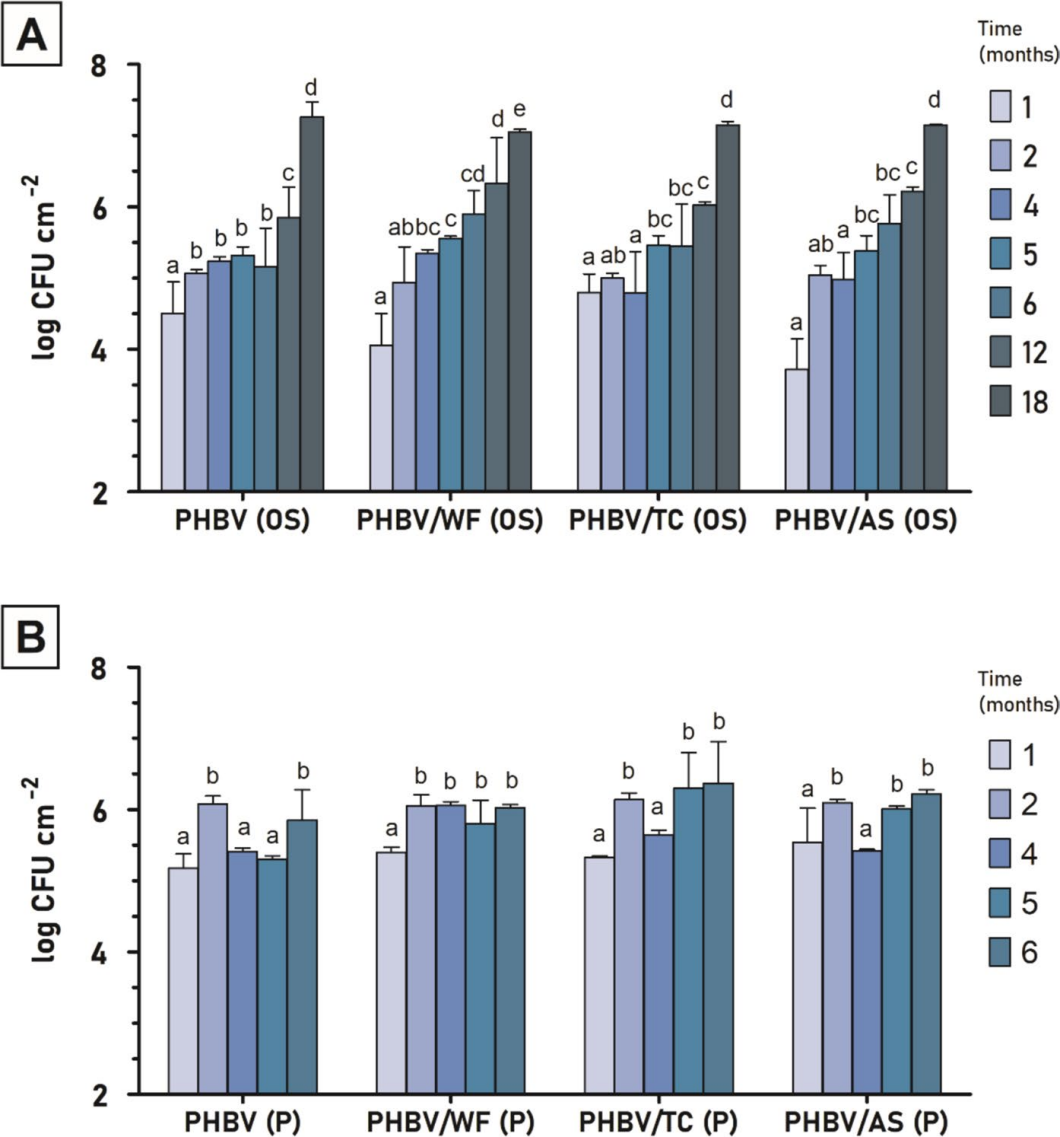
Enumeration of marine heterotrophic cultivable bacteria on PHBV and its composites exposed to marine conditions in **A** open sea and **B** port waters. Different letters (**a**–**d**) within the same material group of bars indicate that there are significant differences (p < 0.05) among them due to the exposure time (to the least significant difference (LSD) test according to one-way ANOVA analysis)

Results of counts of cultivable heterotrophic marine bacteria adhered to the surface of the different 500 µm-materials are shown in Fig. 3. The statistical analysis revealed different patterns depending on the environment where the exposure took place.

As evidenced in Fig. 3A in OS, no differences were observed among compositions. Only PHBV/AS composite presented a significantly lower bacterial surface density (BSD) (p < 0.005) in the first month. This suggested that, in general, neither fiber incorporation nor the type of fiber had a substantial impact on bacterial adhesion and development. These small differences are coherent with the minor variations in terms of mass loss (see Sect. "Mass loss: disintegration") between the neat polymer and composites of 500 µm thickness. Analysis of the time effect on each sample revealed that the BSD increased with longer exposure times, consistent with the growth of microbial biofilms on the plastic surface. The average BSD initially ranged from 3.72 to 4.80 log CFU·cm^−2^ after one month, escalating to between 7.05 and 7.26 log CFU·cm^−2^ by month 18. For neat PHBV, significant differences emerged primarily between the first two months, and then between months 6 to 12 and months 12 to 18. In contrast, composites like PHBV/WF, PHBV/TC, and PHBV/AS showed less marked changes during the first 6 months, with the most notable shifts occurring after prolonged immersion. Bacterial counts within the same range were previously described in OS environment by our group (Marín et al. 2023, 2025) and also are comparable with those reported under marine laboratory conditions (Dussud et al. 2018a; Morohoshi et al. 2018).

Unlike observations in the OS environment, fiber incorporation into the PHBV matrix in the P environment significantly influenced bacterial adhesion on the material surfaces during intermediate exposure periods (refer to Fig. 3B). No significant differences in composition effects were noted at months 1 and 2 (p > 0.005), but distinct variations became apparent at months 4 and 5. By month 4, PHBV/WF and PHBV/TC demonstrated notably higher BSD (p < 0.005) compared to plain PHBV, a trend that persisted into month 5. The exposure time effect showed smaller differences compared to the OS conditions, likely due to the shorter exposure duration. While PHBV alone did not exhibit a consistent BSD trend over time, the addition of the three fibers resulted in an overall increase in bacterial accumulation with extended immersion time.

Additional statistical analysis consisting of a two-way ANOVA (variables: fiber incorporation/type of fiber and exposure time) and a three-way ANOVA (variables: fiber incorporation/type of fiber, exposure time, and environment) were performed (see Table S1 in Supplementary Material). These analyses confirmed that the incorporation of fibers to PHBV had an impact on bacterial adhesion in P waters, but not in OS. When the effect of the environment was analyzed, it was concluded that the P location led to a higher bacterial accumulation compared to OS location, probably due to the proximity of human activities in the port. This finding aligns with the disintegration results (Sect. "Mass loss: disintegration") which revealed a higher mass loss in P than in OS. Taken together, these results underscore the influence of the location of the plastics in the sea on the number of microorganisms adhered to their surface, which may have a differential impact on their biodegradation behavior.

### Surface roughness: SEM

**Fig. 4:**
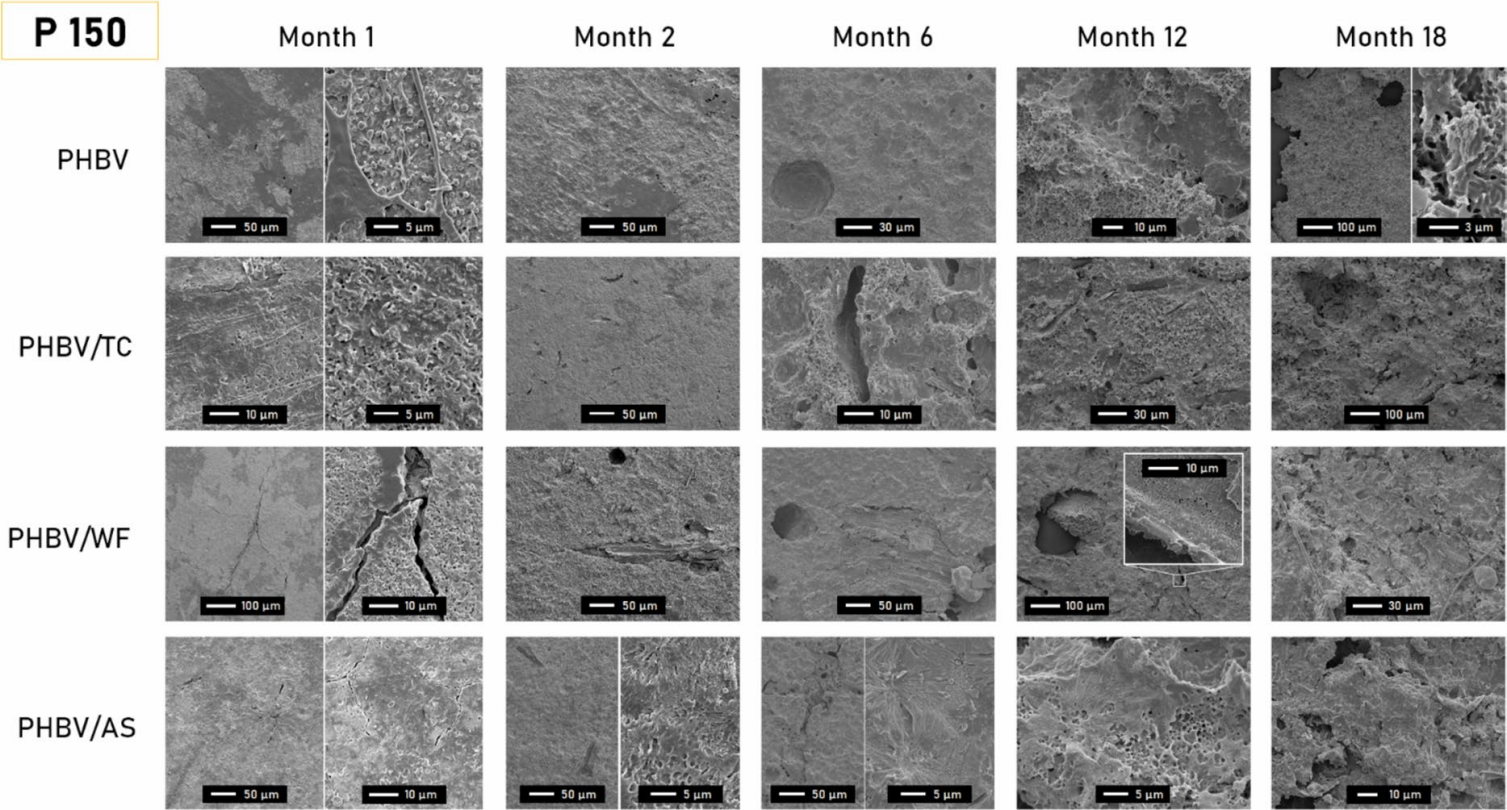
Evolution of surface topography of 150 µm-materials after 1, 2, 6, 12, and 18 months of immersion in the port (P) environment. For sake of clarity, some pictures are shown with two different magnification scales

**Fig. 5:**
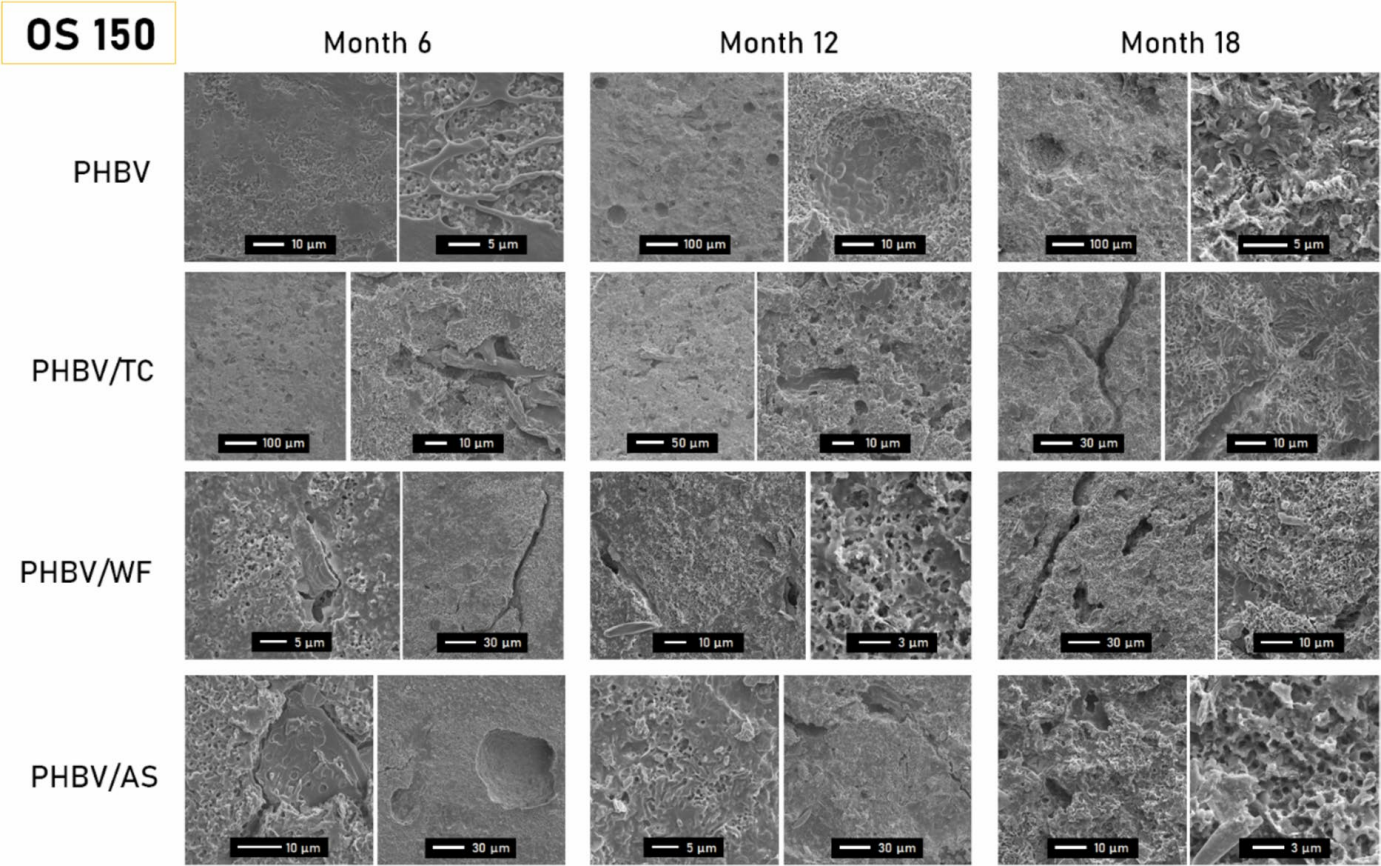
Evolution of surface topography of 150 µm-materials after 6, 12 and 18 months of immersion in the open sea (OS) environment. For sake of clarity, some pictures are shown with two different magnification scales

The evolution of the surface structure over 18 months of immersion was characterized by SEM. Taking P150 samples as a reference, the influence of the environment, thickness, and fiber on the surface degradation of the materials can be appreciated (Figs. 4 and 5).

In all compounds, the PHBV surface transitioned from an uneven mix of degraded and intact areas in the first month to uniformly eroded by month 18. By month 6, spherical cavities ranging from 10 to 50 µm were visible, evolving into larger pores over time. Subsequently, some cavities developed sharper edges, characteristic of crystalline structures, suggesting a microbial preference for the amorphous phase of the polyester (Rutkowska et al. 2008). This phenomenon was particularly noticeable in PHBV/AS at months 6 and 12, where the crystalline spherulites were distinctly visible.

According to SEM observations, the addition of fibers seemed to promote disintegration independently of the type of fiber. Especially in the first 2 months, the degradation of the surface seemed to be rougher and more homogeneous in composites than in neat PHBV. This could be due to the convergence of several reasons. On the one hand, materials seemed to easily break along the edges of the particles embedded. The tension at the fiber-matrix interface may lead to long cracks as the polymer phase weakens by weathering and/or biodegradation (Sethi and Ray 2015; Brebu 2020). As shown in the micrographs of M1, the larger the particle size, the larger the cracks (PHBV/WF > PHBV/AS > PHBV > TC). On the other hand, the hydroxylated components of the lignocellulosic fillers (hemicellulose and cellulose) could link water molecules by hydrogen bonds helping moisture and extracellular enzymes to penetrate the hydrophobic structure of PHBV, thus boosting the biodegradation process (Hammiche et al. 2020). Moreover, the absorbed water in porous structures such as WF or AS could have swollen up the fibers, increasing the pressure and therefore, mechanically fragmenting the surrounding polymer (Hammiche et al. 2020; Brebu 2020).

The increasing number of fiber-shaped cavities in SEM micrographs suggested the faster degradation of the polymer, as the contact surface of the material with the biofilm increases leading to higher mass loss. Isolated signs of fiber degradation were found at month 12 for PHBV/WF, in contrast to the signs found in our previous study in composting conditions (Feijoo et al. 2023). Although this was not a general trend, it may prove that is possible. However, the unbalanced degradation rate of both components (matrix and fillers) of the composites results in no enhancement of global biodegradation, including mineralization (Meereboer et al. 2020b).

Surface degradation patterns (cracks, holes, roughness) in 150 µm-samples submerged in a port environment lagged by six months compared to those in the open sea, as seen in Fig. 5. For example, the degradation observed in open sea samples at month 6 mirrored what was seen in port samples at month 1 or 2. This slower biodegradation rate in the open sea could stem from a lower microorganism concentration and other abiotic factors like reduced light exposure (1 m-depth in port vs 20 m-depth in open sea) or pollution (Wang et al. 2021; Meereboer et al. 2020a). A high concentration of organic matter like in a port environment, could result in anaerobic conditions that are usually more favorable for PHBV biodegradation (Bátori et al. 2018).

For 500 µm-samples in both open sea and port environments, detailed in Supplementary Material (Figures S1 and S2), SEM revealed erosion despite the absence of visible macroscopic changes. In the open sea samples (OS500), they resembled the 150µmm samples without significant differences, but with some AS fiber degradation evident by month 6, suggesting the action of opportunist microorganisms (Du et al. 2022). In the port (P500), where degradation was quicker, a pattern emerged where 12-month micrographs mirrored those at much earlier times, indicating a layer-by-layer degradation process. This confirms PHBV’s biodegradation from the surface inward, with PHBV/AS at 12 months showing similarities to 6-month samples, highlighting a consistent biodegradation mechanism (Meereboer et al. 2020a; Dilkes-Hoffman et al. 2019).

### Surface roughness: profilometry

**Fig. 6:**
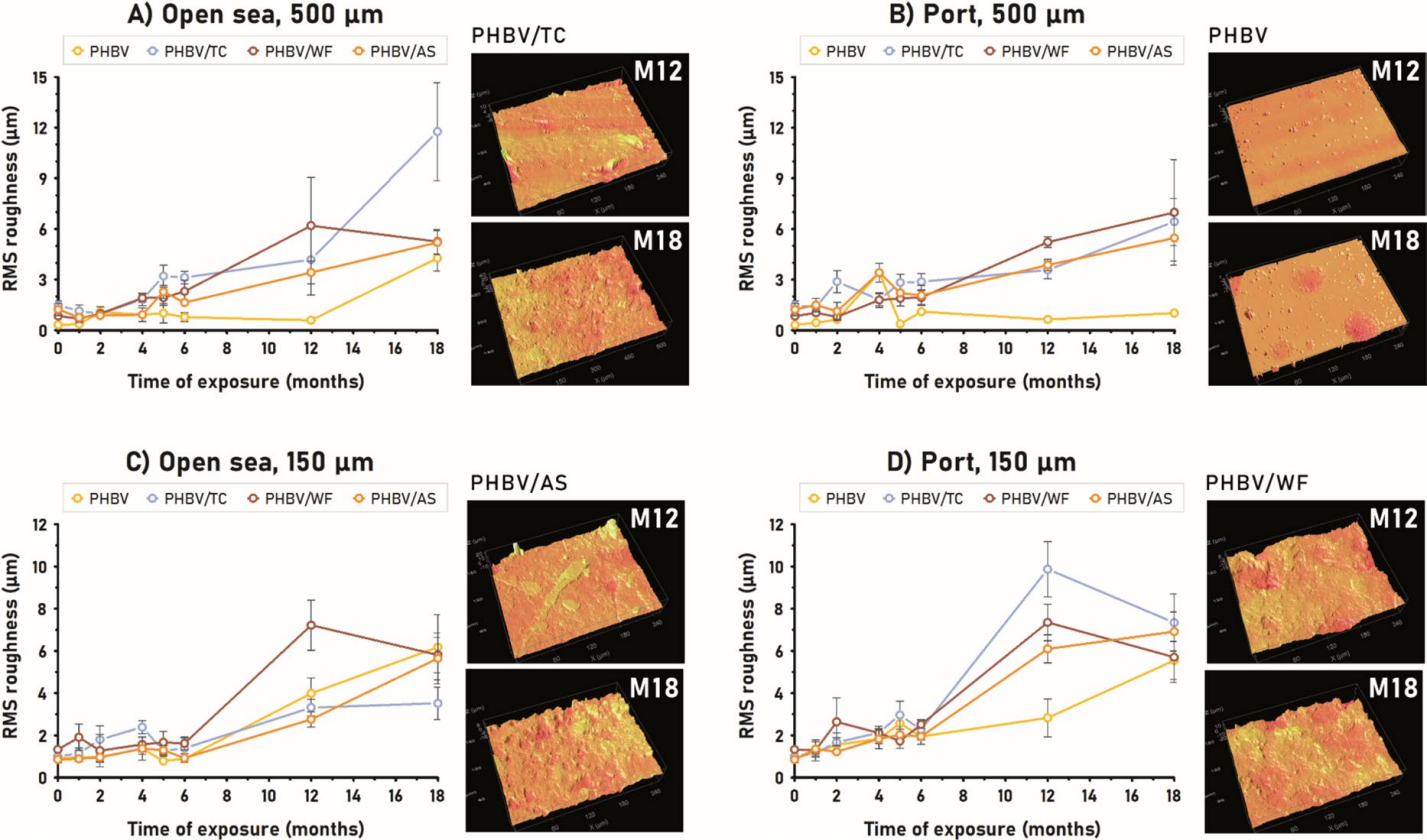
Roughness (RMS) of PHBV and its composites over exposure time to seawater under the four conditions studied (environment—thickness): **A** Open sea 500 µm, **B** Port 500 µm, **C** Open sea 150 µm, and D) Port 150 µm. On the right of each plot, a selection of 3D images of the surfaces of the materials at months 12 and 18 are displayed

Surface roughness, defined as the average amplitude between peaks and valleys, is a parameter closely related to biofilm formation and biodegradation (Song et al. 2015; Dussud et al. 2018b). Although surface topography, which is the pattern distribution of those peaks and valleys, is also an important factor, a rougher surface generally results in a stronger bacterial adhesion to the material (Song et al. 2015). During biodegradation, the microbial attack modifies the surface roughness, which may promote further biofilm density by increasing contact area microbe-material and thus, raise the progress of biodegradation (Dussud et al. 2018b; Nishida and Tokiwa 1993). The variation of surface roughness (RMS) over immersion time in every marine environment, is shown in Fig. 6. The addition of fibers to PHBV led to rougher initial surfaces. In 500 µm-samples, PHBV/TC showed the highest RMS (1.47 µm) closely followed by PHBV/AS and PHBV/WF, while PHBV presented the smoothest surface (0.32 µm) prior to immersion. In 150 µm-samples, PHBV/WF, where the particles are larger than the thickness of the specimen, showed the highest RMS (1.33 µm). The initial RMS values of the rest of the materials ranged from 0.86 to 1.01 µm.

Over the whole 18-month study, all materials experienced increasing RMS roughness as biodegradation progressed. Within a global increasing trend, all materials exhibited periodical increasing–decreasing fluctuations of RMS similar to those reported by Dussud et al. (Dussud et al. 2018b) for PHBV. For instance, the surface roughness of PHBV/WF reached a maximum of 2.64 µm at month 2 which reduced to 1.73 µm at month 5. From this valley, RMS increased to 7.35 µm, a new maximum peak at month 12. Similar examples can be found for PHBV/TC in OS150 from month 1 to 5, or for PHBV/AS and PHBV in P500 from month 2 to month 5. These cyclic modifications of the RMS roughness seemed to be a reflection of a layer-by-layer degradation pattern. The microorganisms would increase and decrease the height of peaks and valleys of the surface before continuing to the internal layers of the material.

In a general overview, all composites showed higher RMS values than PHBV over time. Despite that microbial attack increased the amplitude between peaks and valleys, fibers (or their spots) supposed an additional level of roughness. However, no clear fiber-type effect was found regarding the specific environment. In OS150 and P500, PHBV/WF showed the highest RMS values while in P150 and OS500 the highest RMS values were reached by PHBV/TC. In any case, PHBV/AS showed lower surface roughness than the other two biocomposites. This difference could be ascribed to the shape (L/D ratio) and particle size of TC and WF, respectively.

### Mass loss: disintegration

**Fig. 7:**
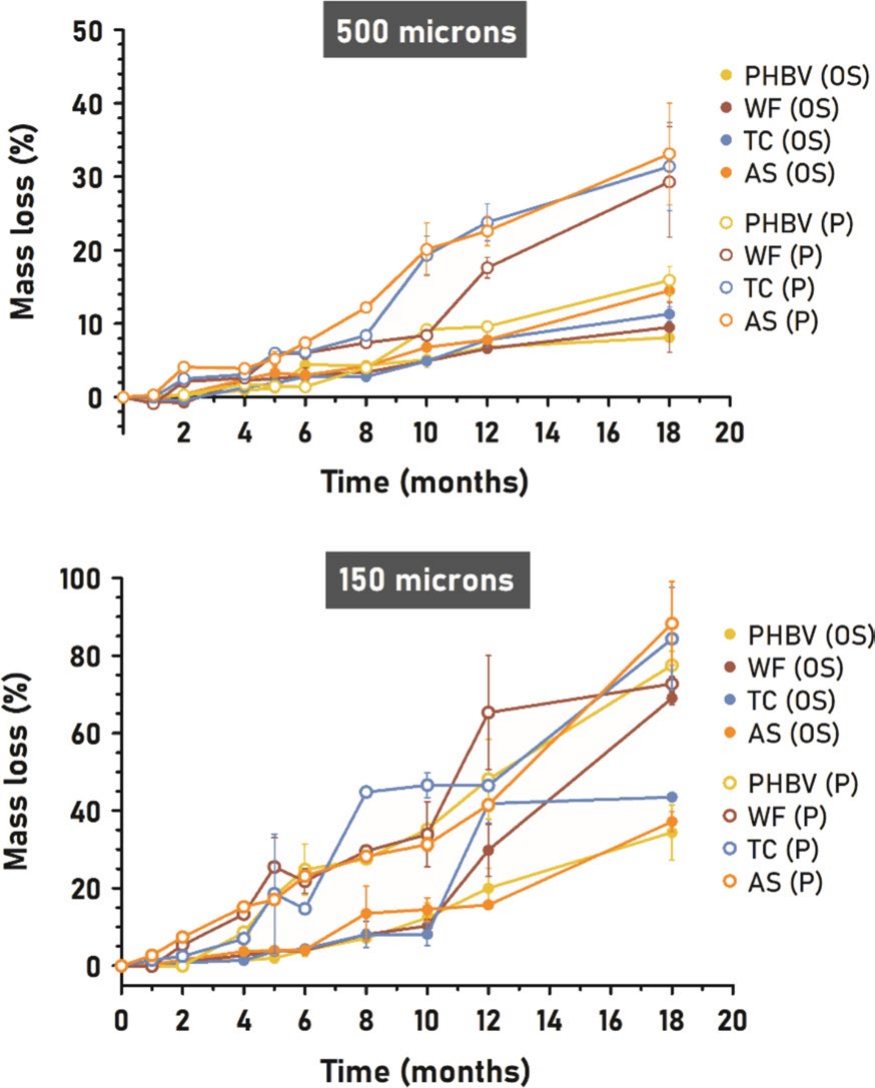
Average disintegration results (D%) over 18 months of seawater exposure of 500 µm-materials (up) and 150 µm-materials (down) in both marine environments

Biodegradation is a complex process that involves three main steps: 1) formation of biofilm, 2) fragmentation, and 3) mineralization (Dilkes-Hoffman et al. 2019; Maity et al. 2021). The disintegration of aliphatic polyesters like PHBV takes place via enzymatic hydrolysis: the extracellular enzymes from microorganisms break down complex molecules into short-chain ones (e.g. oligomers, dimers, monomers) that can be assimilated by bacterial cells (Meereboer et al. 2020a; Maity et al. 2021). Nonetheless, the loss of integrity of materials can be caused also by abiotic factors such as light (photodegradation) or mechanical forces (waves in the case of the marine environment) (Dilkes-Hoffman et al. 2019; Brebu 2020; Gewert et al. 2015). Every mass loss was included in the percentage of disintegration (D%) regardless of its origin. Figure 7 shows the progress of disintegration of PHBV and its composites over 18 months of immersion in each environment (port and open sea, 150 and 500 µm thickness).

As expected, thinner samples reached higher overall D% values at the end of the experiment, closely proportional to the thickness. For instance, PHBV/AS (P) reached 88% of disintegration with 150 µm thickness while it achieved 33% with 500 µm thickness. As previously observed by SEM, the most favorable environment for degradation was the port, where absolute disintegration values were higher for all materials compared to their equivalents in the open sea.

For the sake of comparison between environment and filler type, the overall rate of biodegradation was calculated for all materials, whose values are shown in Table 1. The dynamic biodegradation rate at each immersion time along with the absolute disintegration percentages are summarized in Tables S2 and S3 of Supplementary Material. In general, the biodegradation rate of each thickness material remained similar within the same environment (P150 vs P500, OS150 vs OS500) with the exception of PHBV/TC and PHBV/WF in port, which rates were 0.015 and 0.010 mg·day^−1^·cm^−2^ higher in 500 µm samples. Nonetheless, it could be seen that the biodegradation rate of each material in the open sea is duplicated or triplicated in the port. This could be attributed not only to different microbial diversity but also to the organic-richer waters of a port which enhance the enzymatic function (Meereboer et al. 2020a). Port samples usually were covered by sediments and biofouling, which create an anaerobic environment also favorable for PHA degradation (Federle et al. 2002). When comparing the biodegradation kinetics obtained in this study (0.008–0.039 mg day⁻^1^ cm⁻^2^) with those reported for PHA in marine environments (0.04–0.09 mg day⁻^1^ cm⁻^2^, (Dilkes-Hoffman et al. 2019)), slightly lower values were observed. This deviation can be ascribed to the lower hydroxyvalerate (HV) content and the relatively higher crystallinity of the PHBV grade studied, compared with the materials evaluated in that work, both factors known to hinder water uptake and enzymatic accessibility, thus slowing down degradation kinetics.

**Table 1:** Biodegradation rate in mg·day^−1^·cm^−2^ of PHBV and its composites in each different environment after 18 months of immersion. The values were normalized to the initial surface area according to the method defined by Dilkes-Hoffman et al. (Dilkes-Hoffman et al. 2019)

	PHBV	PHBV/TC	PHBV/WF	PHBV/AS
OS 500	0,008	0,014	0,023	0,012
P 500	0,019	0,039	0,034	0,033
OS 150	0,009	0,016	0,019	0,014
P150	0,018	0,024	0,024	0,029

In both environments, the incorporation of fibers improved the marine disintegration of the polyhydroxyalkanoate. The biodegradation rate of PHBV varied from 0.008–0.009 mg·day^−1^·cm^−2^ to a maximum of 0.019–0.023 mg·day^−1^·cm^−2^ with the addition of WF in open sea. In port, the biodegradation rate of PHBV fluctuated from 0.018–0.019 mg·day^−1^·cm^−2^ to a maximum of 0.029–0.033 mg·day^−1^·cm^−2^ with the addition of AS, even though in this environment the differences between fiber type were low.

Although evidence of fiber degradation was detected by SEM, the isolated nature of this event led to the assumption of a biphasic degradation of composites (Meereboer et al. 2020b). In this case, mass loss is highly influenced by factors that control the fiber detachment from the matrix such as size, shape, and chemical composition. In particular, PHBV/AS reached the highest disintegration percentages at the end of the experiment, closely followed by PHBV/TC and PHBV/WF.

In port, PHBV/TC exhibited the highest biodegradation rate (0.039 mg·day^−1^·cm^−2^) in the case of 500 µm samples whereas PHBV/AS was the faster (0.029 mg·day^−1^·cm^−2^) in the case of for 150 µm samples. Compared to PHBV/WF, the differences can be attributed to the fiber characteristics such as size. The 15–20 times lower size of TC and AS fibers may contribute to their easier detachment (and the corresponding mass loss) due to the lower amount of PHBV that bacteria have to consume around a single particle. Along with lower size, a higher contact area (L/D ratio) between polymer and fiber may promote degradation of the composite by driving moisture to the inner structure of PHBV using fibers as hygroscopic channels. For instance, although the aspect ratio of spherical AS (L/D = 2) is lower than that of WF (L/D = 4), the significantly lower size of AS increases the overall contact area with the polymer, thereby enhancing biodegradation to a greater extent. Consequently, the mass loss of PHBV/WF was slowed down in general, but great peaks of D% appeared at specific times (i.e. from month 10 to 12 in port, both thicknesses) when a batch of WF particles got detached at once.

Exceptionally, WF was the fiber that favored most the degradation of PHBV in OS150 reaching 69% of disintegration in 18 months. In this environment, the water flow may help to the premature break of a composite whose filler particles are larger than the thickness of the material. Once the surrounding PHBV is weakened, WF fiber could easily set loose due to wave action (Sridewi et al. 2006) thus pulling a portion of polymer not completely degraded.

### Surface chemistry: FTIR

**Fig. 8:**
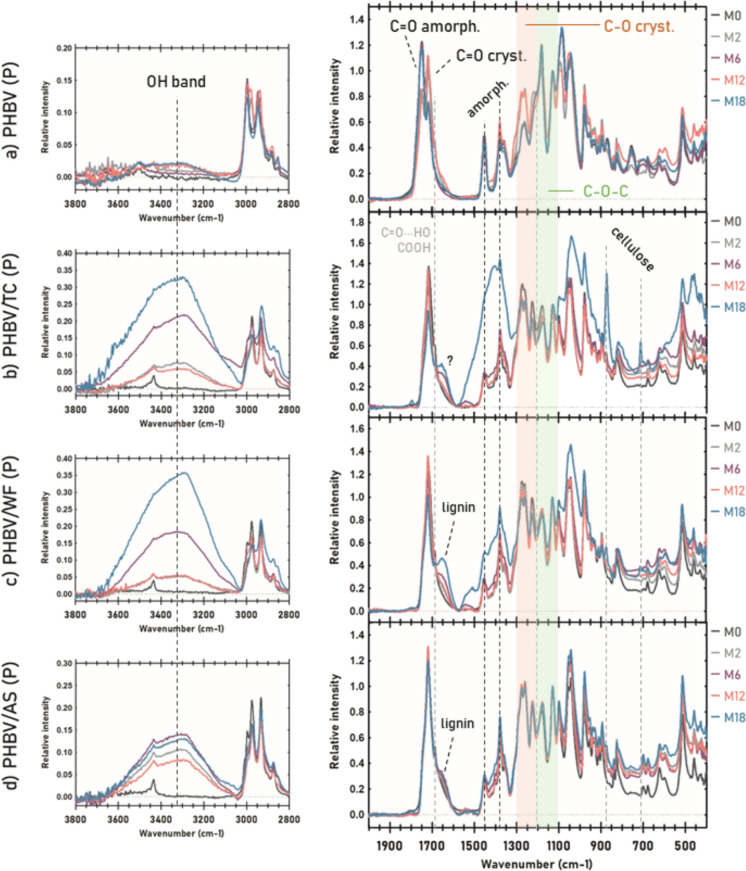
FTIR spectra of 500 µm samples in port environment: **a** PHBV, **b** PHBV/TC, **c** PHBV/WF, and **d** PHBV/AS

**Fig. 9:**
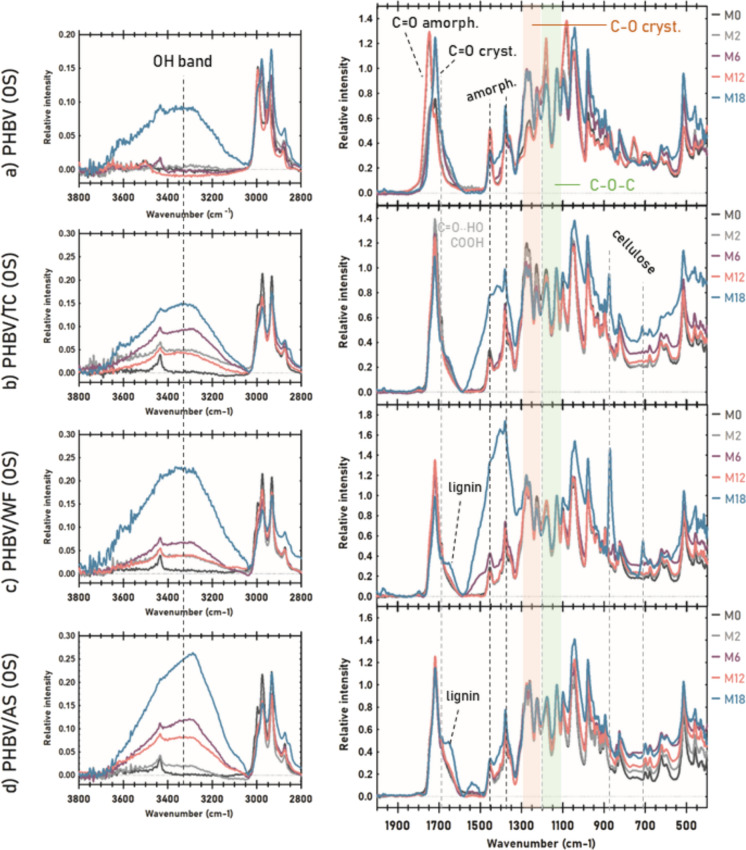
FTIR spectra of 500 µm samples in the open sea: **a** PHBV, **b** PHBV/TC, **c** PHBV/WF, and **d** PHBV/AS

The progress of marine degradation of PHBV and its composites was also studied by FTIR. The spectra of 500 µm materials over immersion time are shown in Figs. 8 and 9 while the spectra of 150 µm samples can be found in Supplementary Material (Figures S3 and S4). For better comparison, spectra were normalized using the peak at 1130 cm^−1^ (C–O–C stretching) as an internal standard. The assignments of the vibrational bands and their intensities can be found in Supplementary Material (Tables S3, S5-S7). In agreement with the research of Xu et al. (Xu et al. 2016), FTIR spectra of the composites were qualitatively similar to the spectrum of the polymer matrix due to the overlapping of most of the spectral bands of each fiber and PHBV. FTIR spectra of the TC, WF and AS fibers are available in Supplementary Material (Table S4). However, the addition of fibers to composites modified the relative intensity of some absorbance bands, indicating changes in the crystalline structure of the polyester.

The wide band centered at 3300 cm^−1^ corresponding to –OH stretching (Voronova et al. 2022), was only noticeable for PHBV at M18 in open sea and very slightly in port as can be seen in Figs. 8a and 9a respectively. Conversely, all composites showed progressive higher intensity of OH band. This is indicative of the cleavage of ester (> C = O) or O-R groups by enzymatic hydrolysis, thus increasing the ratio of terminal hydroxyl groups (Abe and Doi 1999). The breakdown of the ester bonds is also supported by the appearance of a shoulder in the carboxyl peak at 1686 cm^−1^ that was ascribed to the formation of new carboxylic acid end-groups (Izumi and Temperini 2010) as well as to conjugated carbonyl groups by hydrogen bonding (Volova et al. 2022).

In the carbonyl region, two overlapped signals were attributed to C = O stretching of the ester in PHBV matrix: 1745 cm^−1^ corresponding to amorphous domains and 1718 cm^−1^ to crystalline regions. The ratio between the intensity of both peaks (R_1720/1745_) over immersion time is displayed in Table 2. Before exposure to seawater, the amorphous carbonyl peak was the most intense of the PHBV spectra while the crystalline showed approximately half of intensity (R_1720/1745_ = 0.62). In the open sea, this ratio increased up to 2.07 at M6 which seemed to indicate a preferential and progressive degradation of the amorphous phase (Dilkes-Hoffman et al. 2019; Rutkowska et al. 2008). After 6 more months, the crystalline-amorphous ratio returned to the initial stage value (R_1720/1745_ = 0.49). In general, the studied materials showed a cyclic behavior similar to other parameters studied such as surface roughness. According to Abe and Doi (Abe and Doi 1999), the PHA depolymerase binds to the surface of crystalline lamellae, especially at the edges, although the polymer chains with higher mobility like in the amorphous phase are easily attacked by the active site of the enzyme. Hence, once the surrounding amorphous phase is consumed, depolymerases need to degrade the crystalline substrate they are bound to before reaching a new layer of the bulk. In port, the lower difference between R_1720/1745_ over immersion time aligned to mass loss and surface roughness values, points to shorter cycles, suggesting a more aggressive degradation in this environment.

**Table 2:** Carbonyl indexes and crystalline/amorphous ratio for PHBV and its composites (500 µm thickness) calculated according to Antunes et al. (Antunes et al. 2020)

		CRYSTALLINE		AMORPHOUS		Crystall.-amorph. RATIO	
		Carbonyl index 1720/1380		Carbonyl index 1745/1380		1720/1745	
		OS	P	OS	P	OS	P
	M0	1,56		2,53		0,62	
	M2	2,09	1,90	1,15	1,57	1,82	1,21
PHBV	M6	2,06	1,54	1,00	2,55	2,07	0,60
	M12	1,35	1,86	2,74	1,42	0,49	1,31
	M18	1,79	1,65	0,83	2,56	2,17	0,64
	M0	1,93		0,75		2,57	
	M2	2,03	1,90	0,75	0,80	2,71	2,37
PHBV/TC	M6	1,81	1,60	0,71	0,60	2,56	2,66
	M12	1,90	1,90	0,89	0,67	2,15	2,83
	M18	1,10	0,65	0,47	0,25	2,34	2,61
	M0	0,29		0,68		0,42	
	M2	1,94	2,08	0,68	0,75	2,85	2,78
PHBV/WF	M6	1,71	1,74	0,65	0,69	2,62	2,53
	M12	2,01	2,10	0,79	0,77	2,55	2,73
	M18	0,57	1,13	0,20	0,46	2,78	2,45
	M0	0,61		0,77		0,79	
	M2	1,73	1,65	0,70	0,62	2,46	2,66
PHBV/AS	M6	1,75	1,73	0,71	0,66	2,48	2,63
	M12	1,86	1,98	0,72	0,70	2,56	2,82
	M18	1,49	1,58	0,55	0,61	2,70	2,60

In the fingerprint region there were some remarkable variations of PHBV FTIR bands related to molecular ordering. The intensity of 1452 cm^−1^ band (CH_3_ asymmetric bending) assigned to the amorphous phase, and of 1359 cm^−1^ peak (CH_2_ wagging) which is related to the hydroxyvalerate (HV) content (Izumi and Temperini 2010), were reduced during those months when the amorphous index decreased, thus indicating preference of the exo enzymes for degrading less packed polymer chains (Abe and Doi 1999; Izumi and Temperini 2010). The bands ranging from 1210 to 1276 cm^−1^ were attributed to different C–O vibrational modes, all related to the crystalline phase and conformation of the helical chains. The shifting of 1210 up to 1227 cm^−1^ as well as the increase of intensity and doublet of 1260 cm^−1^ with 1276 cm^−1^ peak, suggested a reorganization of the polyester chains as enzymatic degradation progressed. Finally, the bands 1180 and 1083 cm^−1^ associated with C–O–C stretching, significantly decreased their intensity over time which may also confirm the breakdown of ester and O–R bonds.

In the case of biocomposites, bands related to stretching C-O crystalline decreased, while CH3 deformation bands turned into a more intense, wider and not well-defined band. All these changes evidenced enzymatic degradation of PHBV together with hydroxyl band increasing at 3300 cm^−1^ (Voronova et al. 2022; Abbasi et al. 2022; do Nascimento Silva et al. 2021). The presence of fibers appeared to enhance marine degradation of the polyester matrix. In addition, two new peaks appeared in the fingerprint region of all composites: 1) the low-intensity peak at 713 cm^−1^ corresponded to cellulose Iβ allomorph (Hong et al. 2021; Boukir et al. 2019) was only appreciable when the polymer/fiber ratio decreased, and 2) the intense peak at 876 cm^−1^ could be attributed to oxidized cellulose (Lucia et al. 2020) as it can be metabolized by extracellular monooxygenases of fungi and bacteria present in terrestrial and aquatic ecosystems (Cannella et al. 2016).

In the case of PHBV/TC (Figs. 8b and 9b), 897 cm^−1^ band ascribed to C–C stretching of both PHBV and cellulose almost disappeared, evidencing skeletal degradation of the whole composite. Moreover, the amorphous carbonyl index (Table 2) decreased by 3 times in port and by twice in the open sea at the end of the immersion, whereas the crystalline phase was substantially degraded at M6 and M18 more intensely in port environment. In the case of PHBV/WF, the crystalline-amorphous ratio (Table 2). Followed a similar progression in both environments. Within an increasing trend of the 1720/1745 ratio, the amorphous index remained practically invariable until M18, when its FTIR spectra suffered significant modifications (Figs. 8c and 9c) more noticeable in open sea. It is noteworthy the presence of a new band around 1650 cm^−1^ in the composites with lignocellulosic fillers, PHBV/WF and PHBV/AS. Some authors described this peak as lignin characteristic (Xu et al. 2016; Volova et al. 2022; Zhuang et al. 2020) which could be noticeable over time as PHBV was degraded similarly to 713 cm^−1^ band for cellulose. However, Boukir et al. (Boukir et al. 2019) associated this peak with the formation of quinonic acid from lignin photodegradation. Marine degradation was less evident in FTIR for PHBV/AS (Figs. 8d and 9d) than for the other composites. Contrary to disintegration results and similarly to PHBV/WF, the environment with more influence on degradation according to FTIR spectra and the carbonyl indexes (Table 2) was the open sea.

Notwithstanding only lignocellulosic fibers (WF and AS) showed signs of degradation in SEM micrographs, cellulose was the most influenced component by degradation according to FTIR. Taking into account the lower intensity of cellulose peaks in PHBV/WF and PHBV/AS compared to PHBV/TC, lignin seemed to act like a physical barrier against microbial attack (Xu et al. 2016; Boukir et al. 2019).

In 150 µm materials, the same alterations of the spectra were found (see Figures S3 and S4 in Supplementary Material). As expected for thinner samples, the modifications of intensities and new peaks were more pronounced since M2 due to the greater progress of biodegradation. For the same reason, differences between materials were smoother. Aligned with mass loss results, degradation was also appreciable in PHBV/AS composite in both environments. The lack of new bands suggested no other different degradation mechanism than enzymatic hydrolysis.

### Structural deterioration: mechanical properties

**Fig. 10:**
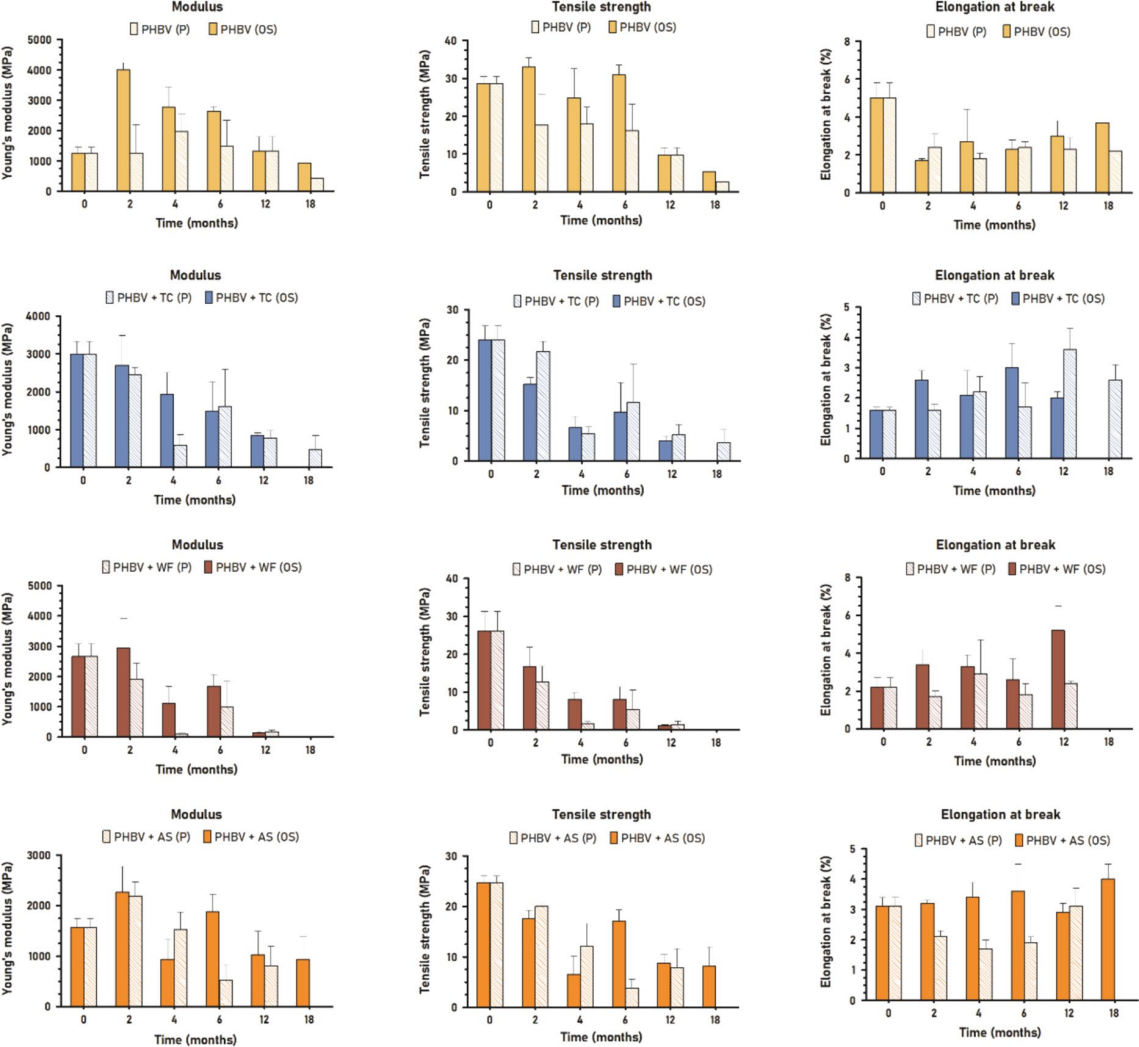
Modulus, tensile strength and elongation at break of 150 µm materials in both environments, open sea and port

Degradation of the structure of materials is also reflected in the alteration of their mechanical properties, particularly tensile strength (Lim and Thian 2022; Gregory and Andrady xxxx). In addition, modulus of elasticity is associated with crystallinity changes while elongation at break is sensitive to defects (Lim and Thian 2022; Feijoo et al. 2022). In the case of fiber-reinforced composites, the tensile strength is more sensitive to the fiber-matrix adhesion whereas the modulus is more dependent on the fiber properties (e.g. size, shape, chemical composition, aspect ratio) whenever a good interaction is achieved (Sánchez-Safont et al. 2018; Hammiche et al. 2020). Materials with a thickness of 150 µm will serve as the benchmark for the mechanical evaluation (Fig. 10). This choice is based on the anticipation of greater deviations according to the more significant deterioration advancement previously revealed.

PHBV is a semicrystalline thermoplastic featuring a relatively high Young’s modulus (1–5 GPa) and low ductility (Kumar et al. 2021; Sánchez-Safont et al. 2021). On top of its inherent fragile character, PHBV experiences gradual embrittlement over time, primarily attributed to the interplay of two phenomena: secondary crystallization and physical aging of the amorphous phase when the temperature exceeds the glass transition temperature (Tg) (Wang et al. 2022), as in seawater conditions. The mechanical properties of PHBV did not follow the same progression in port than in open sea. In OS150, the modulus of elasticity considerably increased from 1300 to 4000 MPa in 2 months. This increment of stiffness could be caused by intrinsic second crystallization, weathering, and/or an increase of crystallinity due to the preferential consumption of amorphous regions by microorganisms (Dilkes-Hoffman et al. 2019; Feijoo et al. 2023). From M2 until the end of the study, the modulus progressively decreased up to 900 MPa. Since SEM observations did not reveal a complete degradation of the amorphous fraction, this reduction seemed to indicate the deterioration of the crystalline regions and the bulk. In P150, modulus of elasticity reached a maximum of 2000 MPa at month 4 which decreased to 400 MPa at month 18. The lower values could indicate a more aggressive degradation in this environment, in which both amorphous and crystalline fractions degrade faster.

Likewise, the tensile strength of PHBV experienced a greater decrease in the port environment. Starting from 28.6 MPa, the parameter was reduced to 5.4 MPa in OS150 and to 2.7 MPa in P150. In the port, tensile strength was reduced by half at M2–M6 while in the open sea the great decrease was at M12. Those decreasing points may indicate when the significant breakdown of the inner structure occurred (Lim and Thian 2022). Regarding elongation at break, it manifested a great decrease at M2 from 5.4% to 1.7% that progressively increased up to 3.7% in OS150. This variation is in agreement with modulus of elasticity and crystalline ratio irrespective of the root origin. In P150, elongation at break decreased and kept ranging from 1.8 to 2.4% over the study, which is aligned with a general and faster degradation of the bulk.

**Fig. 11:**
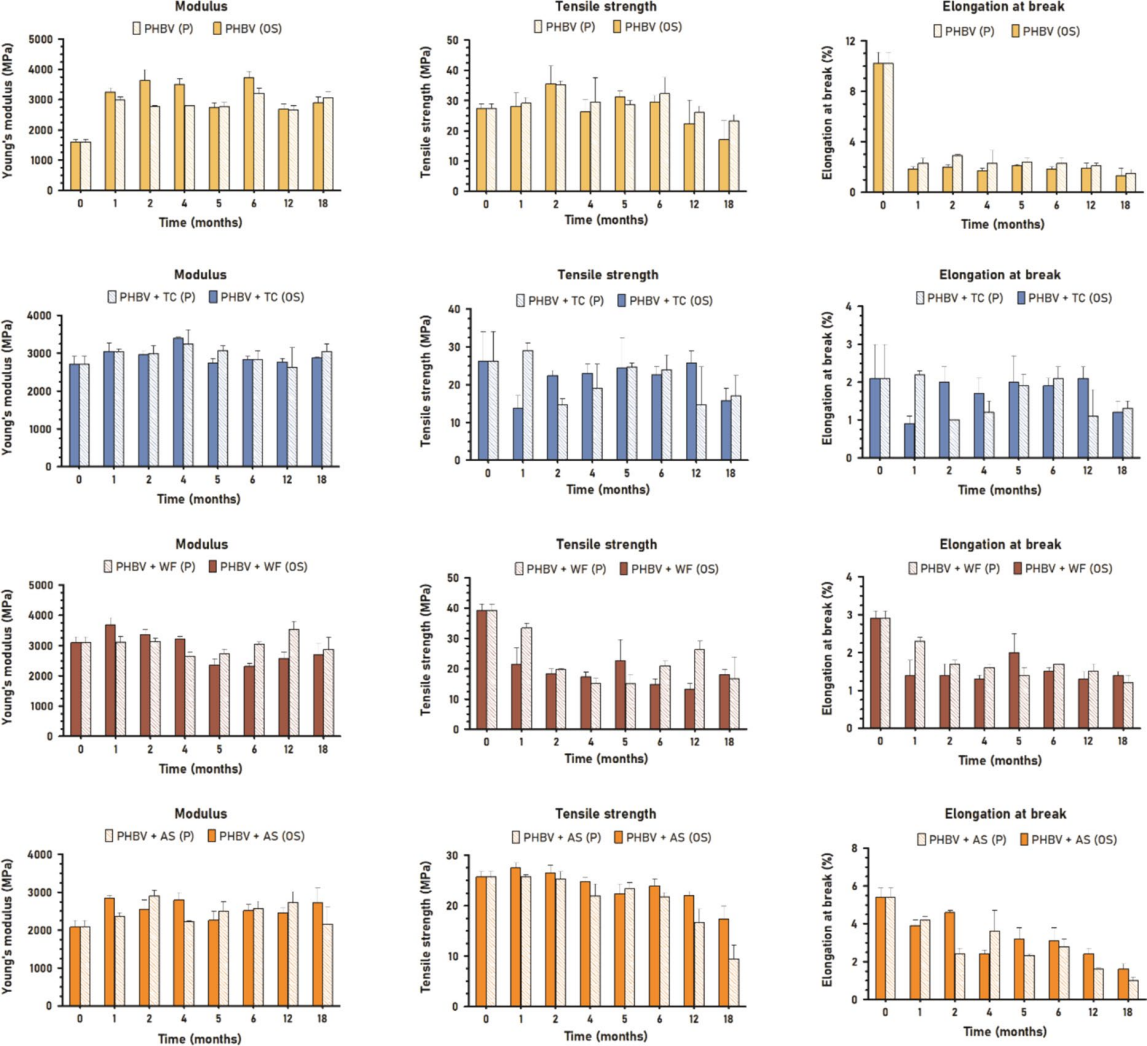
Modulus, tensile strength and elongation at break of 500 µm materials in both environments, open sea and port

As expected, the variations found for 500 µm samples (Fig. 11) were lesser than those for 150 µm thickness. The PHBV-500 modulus of elasticity increased from 1600 to 3200 and 3000 MPa in open sea and port environments respectively. In addition, some increasing–decreasing cycles were observed ranging from 2700 to 3700 MPa (OS500) and 2700–3200 MPa (P500) which seemed to be analogous to RMS cycles. Tensile strength did not suffer any important decrease until month 12, from initial 27.4 MPa to 17.1 MPa (OS500) and 23.2 MPa (P500). This could indicate that previous changes did not affect the bulk, the mechanical properties being preserved despite the embrittlement over time (Dilkes-Hoffman et al. 2019; Lim and Thian 2022). Unlike the other two parameters, elongation at break decreased to around 2% throughout the immersion time. This property being more sensitive to superficial defects, can be highly influenced by other abiotic factors since the first moment of immersion.

The incorporation of fibers implies a reinforcement of the polymer matrix which is manifested in an increase of the modulus of elasticity (Hammiche et al. 2020; Sánchez-Safont et al. 2021). On the other hand, fibers also act as stress concentrators leading to a reduction in elongation at break and tensile strength (Sánchez-Safont et al. 2021).

In the case of PHBV/TC, TC fiber led to the highest reinforcement with initial values of modulus, strength and elongation of 3000 MPa, 24 MPa and 1.6% respectively. In both environments, P150 and OS150, the modulus and tensile strength followed a similar global decreasing trend since the first immersion time. Within this drop, two decreasing-increasing cycles can be observed (M0-M4 and M6-M18) in both parameters which is also aligned with RMS results. The incorporation of TC promoted the degradation of the polymer since the beginning, especially the crystalline fraction. As the ratio of crystallinity was modified, the elongation was modified accordingly which slightly increased to a maximum of 3.0% (OS) and 3.6% (P). In 500 µm samples, the variation of crystallinity seemed to be slighter. Modulus of elasticity ranged from 2700 to 3400 MPa (OS500) and 3200 MPa (P500). Tensile strength rapidly decreased to 13.8 MPa (OS500) at M1 and 14.6 MPa (P500) at M2, and progressively increased to initial values in both environments until a new drop at M18. Elongation at break followed the same trend as tensile strength.

In the case of PHBV/WF, 150 µm initial values of modulus, strength and elongation at break were 2700 MPa, 26.1 MPa and 2.2% respectively. Modulus suffered a drastic decrease to 100 MPa approximately in both environments while tensile strength followed a similar behavior. Compared to TC, the lower fiber-matrix adhesion magnified by degradation and the high particle size of WF seemed to promote the premature failure of the matrix. Elongation at break followed the opposite trend ranging to a maximum of 5.2% (OS) and 2.9% (P). As previously observed by other techniques, the port environment had a higher degradation effect. In 500 µm samples, the large size of WF particles helped to transfer the stress into the bulk of the composite, rapidly promoting its failure. As degradation progressed, tensile strength and elongation at break decreased considerably. Modulus was the less affected parameter.

In the case of PHBV/AS, 150 µm initial values of modulus, strength and elongation at break were 1600 MPa, 24.7 MPa and 3.1% respectively. Analogously to PHBV/TC due to the particle size of the fiber, modulus and tensile strength followed the same increasing–decreasing cyclic behavior (M0-M6-M18 in P, and M0-M4-M12 in OS). However, elongation at break was slightly affected in OS150 ranging from 2.9 to 4.0% whilst in P150 decreased to 1.6% in 4 months and then increased to initial value over immersion time. As well as for the other composites, the effect of degradation in mechanical properties was slighter in 500 µm PHBV/AS. All parameters started to decrease from M12 or M18, this drop being higher in the port environment.

### Lab-simulated biodegradation: CO_2_ evolved

After the disintegration of the materials, biodegradation continues towards mineralization. Small molecules yielded by enzymatic hydrolysis can pass the bacterial membrane to be metabolized (or mineralized) into CO_2_ and water, or into CH_4_ in case of anaerobic conditions (Dilkes-Hoffman et al. 2019; Maity et al. 2021; Manfra et al. 2021).

**Fig. 12:**
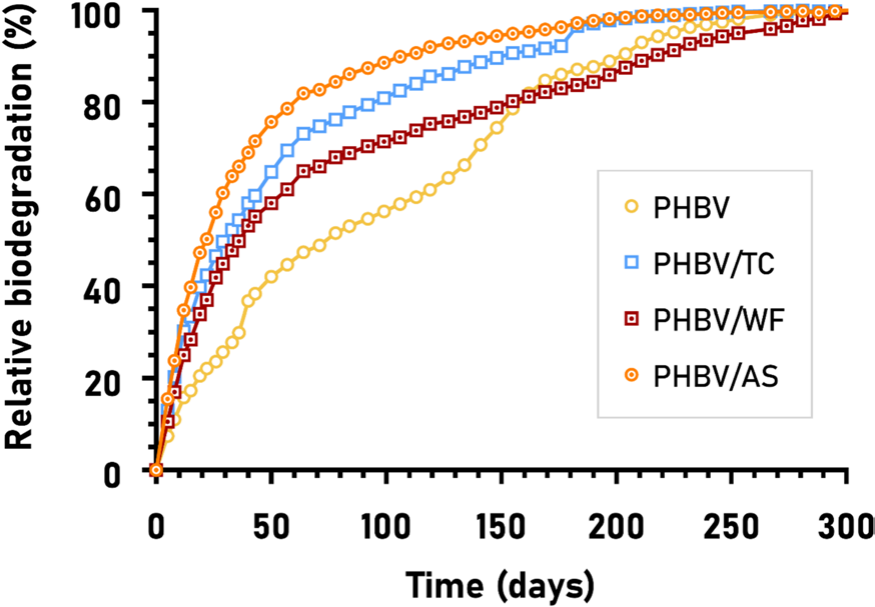
Biodegradation curve (B%) over time of neat PHBV and its composites with purified cellulose (TC), wood flour (WF) and almond shell (AS) in marine lab-simulated conditions

Following ASTM D 6691, measurements of the carbon dioxide evolved by PHBV and its composites in a lab-simulated marine environment were used to estimate their biodegradability behavior in nature. All the materials used in the current study are known to be fully biodegradable in the marine environment (both PHBV and natural lignocellulosic fibers); however, the rate at which this biodegradation takes place, it may change depending on the composition and particular conditions of the sample. Figure 12 shows the relative mineralization or relative biodegradation degree (B%) of PHBV and its composites after 300 days of testing. The samples were used in powdered form trying to replicate the initial conditions of the mineralization step as accurately as possible. At this point, the shape and size of the fibers in composites should not govern their degradation behavior (Meereboer et al. 2020b), however they may play a role in the rate at which the carbon is bioassimilated and mineralized.

Attending to the low cellulolytic capability of the marine microbiome (Meereboer et al. 2021), the presence of fibers may lead to the assumption that the biodegradation rate of the composites would be lower than that of PHBV. Nevertheless, the presence of fibers enhanced the biodegradation kinetics of PHBV.Hence, while neat PHBV reached t₅₀ after 78 days, the composites achieved the same level markedly earlier: at 22 days for PHBV/AS, 30 days for PHBV/TC90, and 35 days for PHBV/WF, thus confirming that the incorporation of lignocellulosic fillers enhanced biodegradation kinetics by approximately 55–70%, with PHBV/AS being the fastest-degrading formulation. This can be readily explained by the fact that cellulose increases the material’s hygroscopicity, facilitating faster and greater water uptake into the polymer matrix. This leads to a higher specific surface area and, consequently, greater accessibility for the extracellular enzymes produced by microorganisms to act on the material (Hammiche et al. 2020; Feijoo et al. 2023). Regarding the influence of fiber type, the mineralization trend closely mirrors that of disintegration, with TC and WF composites switching their relative positions: PHBV/AS > PHBV/TC > PHBV/WF > PHBV. In particular, AS appeared to be the most favorable fiber for promoting PHBV biodegradation. This could be attributed to its highly porous structure, despite the potential inhibitory effect of lignin on water sorption (Meereboer et al. 2020a; Xu et al. 2016).

## Conclusions

Over 18 months, this study evaluated the biodegradation of PHBV-based biocomposites with cellulose (TC), wood flour (WF), and almond shell (AS) fibers versus pure PHBV, in two Mediterranean marine locations: a port and the open sea. Habitat-specific features significantly influenced biodegradation, with the port showing greater bacterial adhesion and higher material weight loss compared to the open sea. Mechanical deterioration (modulus, strength, elongation) was more pronounced in the port, especially after long-term exposure (12/18 months), indicating a significant breakdown of the internal structure of the materials. All lignocellulosic fillers enhanced PHBV biodegradation by improving water sorption and diffusion, with PHBV/AS showing the highest disintegration degree (88% for 150 µm films and 33% for 500 µm sheets) after 18 months. Biocomposite disintegration was primarily influenced by the physical properties of the fibers (size, shape, porosity) rather than their chemical composition, with lignin acting as a barrier to microbial attack. SEM revealed isolated fiber biodegradation signs, confirming the biodegradation of matrix and fiber as biphasic; cellulose and lignin FTIR bands were more noticeable as the polymer/fiber ratio decreased. Mineralization tests under lab-simulated marine conditions reflected the disintegration trends, with AS fibers being the most effective for enhancing PHBV biodegradation rate. This research broadens the limited knowledge on PHBV-based biocomposites biodegradation in marine conditions, aiding in the prediction of lifetime of polymeric consumer products.

## Data Availability

No datasets were generated or analysed during the current study.

## References

[CR1] Abbasi M, Pokhrel D, Coats ER, Guho NM, McDonald AG (2022) Effect of 3-hydroxyvalerate content on thermal, mechanical, and rheological properties of poly(3-hydroxybutyrate-co-3-hydroxyvalerate) biopolymers produced from fermented dairy manure. Polymers. 10.3390/polym14194140 36236088 10.3390/polym14194140 PMC9571417

[CR2] Abe H, Doi Y (1999) Structural effects on enzymatic degradabilities for poly[(R)-3-hydroxybutyric acid] and its copolymers. Int J Biol Macromol 25:185–192. 10.1016/S0141-8130(99)00033-1 10416666 10.1016/s0141-8130(99)00033-1

[CR3] Antunes A, Popelka A, Aljarod O, Hassan MK, Kasak P, Luyt AS (2020) Accelerated weathering effects on poly(3-hydroxybutyrate-co-3-hydroxyvalerate) (PHBV) and PHBV/TiO2 nanocomposites. Polymers. 10.3390/polym12081743 32764247 10.3390/polym12081743 PMC7464598

[CR4] ASTM D6691–17. “Standard Test Method for Determining Aerobic Biodegradation of Plastic Materials in the Marine Environment by a Defined Microbial Consortium or Natural Sea Water Inoculum”. Standard, ASTM International.

[CR5] Bátori V, Akesson D, Zamani A, Taherzadeh MJ, Sárvári Horváth I (2018) Anaerobic degradation of bioplastics: a review. Waste Manag 80:406–413. 10.1016/j.wasman.2018.09.040 30455023 10.1016/j.wasman.2018.09.040

[CR6] Boukir A, Fellak S, Doumenq P (2019) Structural characterization of *Argania spinosa* Moroccan wooden artifacts during natural degradation progress using infrared spectroscopy (ATR-FTIR) and X-ray diffraction (XRD). Heliyon. 10.1016/j.heliyon.2019.e02477 31687572 10.1016/j.heliyon.2019.e02477 PMC6819844

[CR7] Brebu M (2020) Environmental degradation of plastic composites with natural fillers—a review. Polymers. 10.3390/polym12010166 31936374 10.3390/polym12010166 PMC7022390

[CR8] Cannella D, Möllers KB, Frigaard NU, Jensen PE, Bjerrum MJ, Johansen KS, Felby C (2016) Light-driven oxidation of polysaccharides by photosynthetic pigments and a metalloenzyme. Nat Commun 7:11134. 10.1038/ncomms11134 27041218 10.1038/ncomms11134 PMC4822002

[CR9] Cheng J, Eyheraguibel B, Jacquin J, Pujo-Pay M, Conan P, Barbe V, Hoypierres J, Deligey G, Ter Halle A, Bruzaud S, Ghiglione JF, Meistertzheim AL (2022) Biodegradability under marine conditions of bio-based and petroleum-based polymers as substitutes of conventional microparticles. Polym Degrad Stab 206:110159. 10.1016/j.polymdegradstab.2022.110159

[CR10] Chinaglia S, Tosin M, Degli-Innocenti F (2018) Biodegradation rate of biodegradable plastics at molecular level. Polym Degrad Stab 147:237–244. 10.1016/j.polymdegradstab.2017.12.011

[CR11] da Silva AMB, Bercini Martins A, Campomanes Santana RM (2021) 10 - Biodegradability studies of lignocellulosic fiber reinforced composites. In Fiber Reinforced Composites, Woodhead Publishing

[CR12] Das S, Mangwani N (2015) Ocean acidification and marine microorganisms: responses and consequences. Oceanologia 57:349–361. 10.1016/j.oceano.2015.07.003

[CR13] Deroiné M, César G, Le Duigou A, Davies P, Bruzaud S (2015) Natural degradation and biodegradation of poly(3-hydroxybutyrate-co-3-hydroxyvalerate) in liquid and solid marine environments. J Polym Environ 23:493–505. 10.1007/s10924-015-0736-5

[CR14] Dilkes-Hoffman LS, Lant PA, Laycock B, Pratt S (2019) The rate of biodegradation of PHA bioplastics in the marine environment: a meta-study. Mar Pollut Bull 142:15–24. 10.1016/j.marpolbul.2019.03.020 31232288 10.1016/j.marpolbul.2019.03.020

[CR15] do Nascimento Silva R, da Carneiro Silva LR, de Lemos Morais AC, Soares Alves T, Barbosa R (2021) Study of the hydrolytic degradation of poly-3-hydroxybutyrate in the development of blends and polymeric bionanocomposites. J Thermoplast Compos Mater 34:884–901. 10.1177/0892705719856044

[CR16] Du Y, Liu X, Dong X, Yin Z (2022) A review on marine plastisphere: biodiversity, formation, and role in degradation. Comput Struct Biotechnol J 20:975–988. 10.1016/j.csbj.2022.02.008 35242288 10.1016/j.csbj.2022.02.008 PMC8861569

[CR17] Dussud C, Meistertzheim A, Conan P, Pujo-Pay M, George M, Fabre P, Coudane J, Higgs P, Elineau A, Pedrotti M, Gorsky G, Ghiglione J (2018a) Evidence of niche partitioning among bacteria living on plastics, organic particles and surrounding seawaters. Environ Pollut 236:807–816. 10.1016/j.envpol.2017.12.027 29459335 10.1016/j.envpol.2017.12.027

[CR18] Dussud C, Hudec C, George M, Fabre P, Higgs P, Bruzaud S, Delort AM, Eyheraguibel B, Meistertzheim AL, Jacquin J, Cheng J, Callac N, Odobel C, Rabouille S, Ghiglione JF (2018b) Colonization of non-biodegradable and biodegradable plastics by marine microorganisms. Front Microbiol. 10.3389/fmicb.2018.01571 30072962 10.3389/fmicb.2018.01571 PMC6058052

[CR19] Emadian SM, Onay TT, Demirel B (2017) Biodegradation of bioplastics in natural environments. Waste Manag 59:526–536. 10.1016/j.wasman.2016.10.006 27742230 10.1016/j.wasman.2016.10.006

[CR20] Federle TW, Barlaz MA, Pettigrew CA, Kerr KM, Kemper JJ, Nuck BA, Schechtman LA (2002) Anaerobic biodegradation of aliphatic polyesters: poly(3-hydroxybutyrate-co-3-hydroxyoctanoate) and poly(*ε*-caprolactone). Biomacromol 3:813–822. 10.1021/bm025520w 10.1021/bm025520w 12099827

[CR21] Feijoo P, Samaniego-Aguilar K, Sánchez-Safont E, Torres-Giner S, Lagarón JM, Gámez-Pérez J, Cabedo L (2022) Development and characterization of fully renewable and biodegradable polyhydroxyalkanoate blends with improved thermoformability. Polymers. 10.3390/polym14132527 35808571 10.3390/polym14132527 PMC9269288

[CR22] Feijoo P, Marín A, Samaniego-Aguilar K, Sánchez-Safont E, Lagarón JM, Gámez-Pérez J, Cabedo L (2023) Effect of the presence of lignin from woodflour on the compostability of PHA-based biocomposites: disintegration, biodegradation and microbial dynamics. Polymers. 10.3390/polym15112481 37299280 10.3390/polym15112481 PMC10255095

[CR23] Fracz W, Janowski G, Smusz R, Szumski M (2021) The influence of chosen plant fillers in PHBV composites on the processing conditions, mechanical properties and quality of molded pieces. Polymers. 10.3390/polym13223934 34833232 10.3390/polym13223934 PMC8625057

[CR24] Gewert B, Plassmann MM, MacLeod M (2015) Pathways for degradation of plastic polymers floating in the marine environment. Environ Sci Processes Impacts 17:1513–1521. 10.1039/C5EM00207A 10.1039/c5em00207a 26216708

[CR25] Gregory, M.R.; Andrady, A.L., Degradation of plastics at sea. In *Plastics and the Environment*; John Wiley & Sons, Ltd, 2003; chapter 10, pp. 379–401. 10.1002/0471721557.ch10.

[CR26] Guo Y, Wang L, Chen Y, Luo P, Chen T (2019) Properties of Luffa Fiber Reinforced PHBV Biodegradable Composites. Polymers. 10.3390/polym11111765 31717853 10.3390/polym11111765 PMC6918194

[CR27] Hammiche D, Boukerrou A, Grohens Y, Guermazi N, Arrakhiz FE (2020) Mechanical properties and biodegradation of biocomposites based on poly (hydroxybutyrate-co-valerate) and alfa fibers. J Polym Res. 10.1007/s10965-020-02284-1

[CR28] Ho YH, Gan SN, Tan IKP (2002) Biodegradation of a medium-chain-length polyhydroxyalkanoate in tropical river water. Appl Biochem Biotechnol 102:337–347. 10.1385/ABAB:102-103:1-6:337 12396135 10.1385/abab:102-103:1-6:337

[CR29] Hong T, Yin JY, Nie SP, Xie MY (2021) Applications of infrared spectroscopy in polysaccharide structural analysis: progress, challenge and perspective. Food Chem.: X 12:100168. 10.1016/j.fochx.2021.100168 34877528 10.1016/j.fochx.2021.100168 PMC8633561

[CR30] Hubbe M, Lavoine N, Lucia L, Dou C (2020) Formulating bioplastic composites for biodegradability, recycling, and performance: a review. BioResour 16:2021–2083. 10.15376/biores.16.1.Hubbe

[CR31] Izumi CM, Temperini ML (2010) FT-Raman investigation of biodegradable polymers: poly(3-hydroxybutyrate) and poly(3-hydroxybutyrate-co-3-hydroxyvalerate). Vib Spectrosc 54:127–132. 10.1016/j.vibspec.2010.07.011

[CR32] Koller M, Maršálek L, de Miranda Sousa Dias M, Braunegg G (2017) Producing microbial polyhydroxyalkanoate (PHA) biopolyesters in a sustainable manner. New Biotechnol 37:24–38. 10.1016/j.nbt.2016.05.001 10.1016/j.nbt.2016.05.001 27184617

[CR33] Kumar V, Sehgal R, Gupta R (2021) Blends and composites of polyhydroxyalkanoates (PHAs) and their applications. Eur Polym J 161:110824. 10.1016/j.eurpolymj.2021.110824

[CR34] Lim BKH, Thian ES (2022) Biodegradation of polymers in managing plastic waste — a review. Sci Total Environ 813:151880. 10.1016/j.scitotenv.2021.151880 34826495 10.1016/j.scitotenv.2021.151880

[CR35] Lucia A, Bacher M, van Herwijnen HWG, Rosenau T (2020) A direct silanization protocol for dialdehyde cellulose. Molecules. 10.3390/molecules25102458 32466232 10.3390/molecules25102458 PMC7287999

[CR36] Maio A, Gulino EF, Gammino M, Citarrella MC, Scaffaro R (2025) Photochemical degradation of PLA-based green composites containing waste biomass from *Posidonia oceanica*, *Chamaerops humilis* and *Ailanthus altissima*: a comparative study. Polym Degrad Stab 234:111204. 10.1016/j.polymdegradstab.2025.111204

[CR37] Maity S, Banerjee S, Biswas C, Guchhait R, Chatterjee A, Pramanick K (2021) Functional interplay between plastic polymers and microbes: a comprehensive review. Biodegradation 32:487–510. 10.1007/s10532-021-09954-x 34086181 10.1007/s10532-021-09954-x

[CR38] Manfra L, Marengo V, Libralato G, Costantini M, De Falco F, Cocca M (2021) Biodegradable polymers: a real opportunity to solve marine plastic pollution? J Hazard Mater 416:125763. 10.1016/j.jhazmat.2021.125763 33839500 10.1016/j.jhazmat.2021.125763

[CR39] Marín A, Feijoo P, de Llanos R, Carbonetto B, González-Torres P, Tena-Medialdea J, García-March JR, Gámez-Pérez J, Cabedo L (2023) Microbiological characterization of the biofilms colonizing bioplastics in natural marine conditions: a comparison between PHBV and PLA. Microorganisms. 10.3390/microorganisms11061461 37374962 10.3390/microorganisms11061461 PMC10304962

[CR40] Marín A, Feijoo P, Carbonetto B, González-Torres P, Tena-Medialdea J, García-March JR, Gámez-Pérez J, Cabedo L (2025) Long-term monitoring of biofilm succession unveils differences between biodegradable and conventional plastic materials. Mar Pollut Bull 214:117820. 10.1016/j.marpolbul.2025.117820 40090043 10.1016/j.marpolbul.2025.117820

[CR41] Meereboer KW, Misra M, Mohanty AK (2020a) Review of recent advances in the biodegradability of polyhydroxyalkanoate (PHA) bioplastics and their composites. Green Chem 22:5519–5558. 10.1039/D0GC01647K

[CR42] Meereboer KW, Pal AK, Cisneros-López EO, Misra M, Mohanty AK (2021) The effect of natural fillers on the marine biodegradation behaviour of poly(3-hydroxybutyrate-co-3-hydroxyvalerate) (PHBV). Sci Rep. 10.1038/s41598-020-78122-7 33441581 10.1038/s41598-020-78122-7 PMC7806601

[CR43] Meereboer, K.; Mohanty, A.K.; Misra, M 2020 Biodegradable poly(3-hydroxybutyrate-co-3-hydroxyvalerate) and renewable biomass based composites and marine degradation behaviour. PhD thesis, University of Guelph, USA

[CR44] Morohoshi T, Ogata K, Okura T, Sato S (2018) Molecular characterization of the bacterial community in biofilms for degradation of poly(3-hydroxybutyrate-co-3-hydroxyhexanoate) films in seawater. Microbes Environ 33:19–25. 10.1264/jsme2.ME17052 29386425 10.1264/jsme2.ME17052 PMC5877338

[CR45] Muniyasamy S, Anstey A, Reddy M, Misra M, Mohanty AK (2013) Biodegradability and compostability of lignocellulosic based composite materials. J Renew Mater 1:253–272. 10.7569/JRM.2013.634117

[CR46] Nishida H, Tokiwa Y (1993) Effects of higher-order structure of poly(3-hydroxybutyrate) on its biodegradation. II. Effects of crystal structure on microbial degradation. J Environ Polym Degrad 1:65–80. 10.1007/BF01457654

[CR48] Rangappa SM, Siengchin S, Parameswaranpillai J, Jawaid M, Ozbakkaloglu T (2022) Lignocellulosic fiber reinforced composites: progress, performance, properties, applications, and future perspectives. Polym Compos 43:645–691. 10.1002/pc.26413

[CR49] Rhodes CJ (2018) Plastic pollution and potential solutions. Sci Progr 101:207–260. 10.3184/003685018X15294876706211 30025551 10.3184/003685018X15294876706211 PMC10365157

[CR50] Rivera-Briso AL, Serrano-Aroca A (2018) Poly(3-Hydroxybutyrate-co-3-Hydroxyvalerate): enhancement strategies for advanced applications. Polymers. 10.3390/polym10070732 30960657 10.3390/polym10070732 PMC6403723

[CR51] Rutkowska M, Krasowska K, Heimowska A, Adamus G, Sobota M, Musiol M, Janeczek H, Sikorska W, Krzan A, Žagar E, Kowalczuk M (2008) Environmental degradation of blends of atactic poly[(R,S)-3-hydroxybutyrate] with natural PHBV in Baltic Sea water and compost with activated sludge. J Polym Environ 16:183–191. 10.1007/s10924-008-0100-0

[CR52] Sánchez-Safont EL, Aldureid A, Lagarón JM, Gámez-Pérez J, Cabedo L (2018) Biocomposites of different lignocellulosic wastes for sustainable food packaging applications. Compos Part B Eng 145:215–225. 10.1016/j.compositesb.2018.03.037

[CR53] Sánchez-Safont EL, Aldureid A, Lagarón JM, Gámez-Pérez J, Cabedo L (2021) Effect of the purification treatment on the valorization of natural cellulosic residues as fillers in PHB-based composites for short shelf life applications. Waste Biomass Valorization 12:2541–2556. 10.1007/s12649-020-01192-1

[CR54] Seggiani M, Cinelli P, Balestri E, Mallegni N, Stefanelli E, Rossi A, Lardicci C, Lazzeri A (2018) Novel sustainable composites based on poly(hydroxybutyrate-co-hydroxyvalerate) and seagrass beach-CAST fibers: performance and degradability in marine environments. Materials. 10.3390/ma11050772 29751601 10.3390/ma11050772 PMC5978149

[CR55] Sethi S, Ray BC (2015) Environmental effects on fibre reinforced polymeric composites: evolving reasons and remarks on interfacial strength and stability. Adv Colloid Interface Sci 217:43–67. 10.1016/j.cis.2014.12.005 25578406 10.1016/j.cis.2014.12.005

[CR56] Singh S, Mohanty AK, Sugie T, Takai Y, Hamada H (2008) Renewable resource based biocomposites from natural fiber and polyhydroxybutyrate-co-valerate (PHBV) bioplastic. Compos Part A Appl Sci Manuf 39:875–886. 10.1016/j.compositesa.2008.01.004

[CR57] Song F, Koo H, Ren D (2015) Effects of material properties on bacterial adhesion and biofilm formation. J Dent Res 94:1027–1034. 10.1177/0022034515587690 26001706 10.1177/0022034515587690

[CR58] Sridewi N, Bhubalan K, Sudesh K (2006) Degradation of commercially important polyhydroxyalkanoates in tropical mangrove ecosystem. Polym Degrad Stab 91:2931–2940. 10.1016/j.polymdegradstab.2006.08.027

[CR59] Suzuki M, Tachibana Y, Kasuya Ki (2021) Biodegradability of poly(3-hydroxyalkanoate) and poly(*ε*-caprolactone) via biological carbon cycles in marine environments. Polym J 53:47–66. 10.1038/s41428-020-00396-5

[CR60] Volova TG, Kiselev EG, Baranovskiy SV, Zhila NO, Prudnikova SV, Shishatskaya EI, Kuzmin AP, Nemtsev IV, Vasiliev AD, Thomas S (2022) Degradable poly(3-hydroxybutyrate) – the basis of slow-release fungicide formulations for suppressing potato pathogens. Polymers. 10.3390/polym14173669 36080743 10.3390/polym14173669 PMC9460056

[CR61] Voronova MI, Gurina DL, Surov OV (2022) Properties of poly(3-hydroxybutyrate-co-3-hydroxyvalerate)/polycaprolactone polymer mixtures reinforced by cellulose nanocrystals: experimental and simulation studies. Polymers. 10.3390/polym14020340 35054746 10.3390/polym14020340 PMC8780583

[CR62] Wang GX, Huang D, Ji JH, Völker C, Wurm FR (2021) Seawater-degradable polymers—fighting the marine plastic pollution. Adv Sci 8:2001121. 10.1002/advs.202001121 10.1002/advs.202001121 PMC7788598 33437568

[CR63] Wang Q, Xu Y, Xu P, Yang W, Chen M, Dong W, Ma P (2022) Crystallization of microbial polyhydroxyalkanoates: a review. Int J Biol Macromol 209:330–343. 10.1016/j.ijbiomac.2022.04.018 35398060 10.1016/j.ijbiomac.2022.04.018

[CR64] Wei L, Stark NM, McDonald AG (2015) Interfacial improvements in biocomposites based on poly(3-hydroxybutyrate) and poly(3-hydroxybutyrate-co-3-hydroxyvalerate) bioplastics reinforced and grafted with α-cellulose fibers. Green Chem 17:4800–4814. 10.1039/C5GC01568E

[CR65] Xu J, Xu X, Liu Y, Li H, Liu H (2016) Effect of microbiological inoculants DN-1 on lignocellulose degradation during co-composting of cattle manure with rice straw monitored by FTIR and SEM. Environ Prog Sustain Energy 35:345–351. 10.1002/ep.12222

[CR66] Zhuang J, Li M, Pu Y, Ragauskas AJ, Yoo CG (2020) Observation of potential contaminants in processed biomass using Fourier transform infrared spectroscopy. Appl Sci. 10.3390/app10124345

